# Inhibition of gelatinase B/MMP-9 does not attenuate colitis in murine models of inflammatory bowel disease

**DOI:** 10.1038/ncomms15384

**Published:** 2017-05-31

**Authors:** Magali de Bruyn, Christine Breynaert, Ingrid Arijs, Gert De Hertogh, Karel Geboes, Greet Thijs, Gianluca Matteoli, Jialiang Hu, Jo Van Damme, Bernd Arnold, Marc Ferrante, Séverine Vermeire, Gert Van Assche, Ghislain Opdenakker

**Affiliations:** 1 Laboratory of Immunobiology, Department of Microbiology and Immunology, Rega Institute for Medical Research, KU Leuven, Leuven 3000, Belgium; 2 Translational Research Center for Gastrointestinal Disorders (TARGID), Department of Clinical and Experimental Medicine, KU Leuven, Leuven 3000, Belgium; 3 Laboratory of Clinical Immunology, Department of Microbiology and Immunology, KU Leuven, Leuven 3000, Belgium; 4 Faculty of Medicine and Life Sciences, Hasselt University, Hasselt 3500, Belgium; 5 Translational Cell and Tissue Research, Department of Imaging and Pathology, KU Leuven, Leuven 3000, Belgium; 6 Key Laboratory of Modern Chinese Medicines, Ministry of Education, China Pharmaceutical University, Nanjing, Jiangsu 211198, China; 7 Laboratory of Molecular Immunology, Department of Microbiology and Immunology, Rega Institute for Medical Research, KU Leuven, Leuven 3000, Belgium; 8 Department of Molecular Immunology, German Cancer Research Center (DKFZ), Heidelberg 69120, Germany; 9 University Hospitals Leuven, Department of Gastroenterology and Hepatology, Leuven 3000, Belgium

## Abstract

One third of patients with inflammatory bowel disease (IBD) inadequately respond to anti-TNF treatment and preclinical data suggest that matrix metalloproteinase-9 (MMP-9) is a novel therapeutic target. Here we show that IBD clinical and histopathological parameters found in wild type mice challenged with three different models of colitis, acute and chronic dextran sodium sulphate (DSS), and acute 2,4,6-trinitrobenzenesulfonic acid-induced colitis are not attenuated in MMP-9 knockout mice. We find similar colonic gene expression profiles in wild type and MMP-9 knockout mice in control and acute DSS conditions with the exception of eleven genes involved in antimicrobial response during colitis. Parameters of chronic colitis are similar in wild type and MMP-9 knockout mice. Pharmacological inhibition of MMP-9 with bio-active peptides does not improve DSS colitis. We suggest that MMP-9 upregulation is a consequence rather than a cause of intestinal inflammation and we question whether MMP-9 represents a disease target in IBD.

## Abstract (web summary)

Metalloproteinase-9 has been suggested as therapeutic target to treat inflammatory bowel disease. Here de Bruyn *et al*. show that genetic and pharmacological inhibition of metalloproteinase-9 does not ameliorate inflammation and fibrosis in mice challenged with acute and chronic colitis protocols.

Inflammatory bowel diseases (IBD), including Crohn's disease (CD) and ulcerative colitis (UC), are chronic relapsing-remitting diseases of the gastrointestinal tract^1^. Patients present with (bloody) diarrhoea, abdominal cramping, fever, fatigue and unintended weight loss. Both UC and CD are common multifactorial diseases that cause enormous patient discomfort and high healthcare costs for the society. The incidence and prevalence of IBD are increasing worldwide, including in developing countries^2^. Despite extensive research, the etiopathogenesis of IBD is not yet fully understood. It is thought that an abnormal immune response is elicited towards the luminal microbiota in a genetically susceptible host^1^. The introduction of anti-tumour necrosis factor biologicals more than 15 years ago had a major impact on the treatment of IBD patients. Therapeutic goals evolved from symptomatic remission to mucosal healing and lowered hospitalization and surgery rates. However, up to one third of patients become therapy-resistant and, consequently, new pharmacological targets are needed. Within the matrix metalloproteinase (MMP) family, gelatinase B or MMP-9 is suggested as a novel therapeutic target for the treatment of IBD, because MMP-9 expression is associated with disease development and is reduced by efficient treatment, as recently reviewed^3^. Moreover, the covalent complex of MMP-9 with neutrophil gelatinase B-associated lipocalin is a serum marker of mucosal healing in UC^4^ and CD^5^. Animal studies have been conducted to investigate the causal role of MMP-9 in experimental colitis. Single MMP-9^−/−^ and double MMP-2^−/−^/MMP-9^−/−^ mice were claimed to be resistant to the development of acute colitis induced by dextran sodium sulphate (DSS)^6^,^7^,^8^ and monoclonal antibodies against MMP-9 were used to block acute DSS-induced colitis in mice^9^,^10^. On these bases, clinical phase 1 studies in UC patients were completed with an MMP-9 inhibitory antibody (GS-5745, Gilead Sciences)^11^. However, recently, phase 2/3 clinical studies in UC patients (TRIUMPH Study GS-US-326–1100) were terminated after futility and efficacy analyses.

In the present study, we revert and complement published data on MMP-9 gene deficiency in three animal models of colitis: an acute and a chronic DSS-mediated model of colonic inflammation mimicking various aspects of UC and an acute 2,4,6-trinitrobenzenesulfonic acid (TNBS)-mediated model that more closely resembles CD. In addition, we use two peptide inhibitors with proven efficacy towards MMP-9 in an acute DSS-induced colitis model in three different set-ups: multiple dose prophylactic and therapeutic schemes, and continuous infusion via osmotic pumps. We find no differences in clinical or histopathological parameters after genetic or pharmacological inhibition of MMP-9. Therefore, our findings suggest that MMP-9 upregulation is a consequence of the inflammatory process and unlikely represents a therapeutic target in IBD.

## Results

### Genetic background and microbiota of MMP-9^−/−^ and WT mice

MMP-9^−/−^ mice and their wild type (WT; C57BL/6J) littermates were backcrossed for 13 generations and reared under specific pathogen-free (SPF) conditions for more than 15 years within the same insulator (Supplementary Fig. 1). Genetic background characterization was performed on a panel of 1,449 single nucleotide polymorphisms (SNPs) in both MMP-9^−/−^ and WT mice. MMP-9^−/−^ and WT mice were 99.86% and 99.97% of C57BL/6J recipient genome, respectively. Two SNPs (rs3664408 and rs13476889) of 129S6/Sv background could be discriminated in the MMP-9^−/−^ mice at chromosome 2, related to the region of genetic modification for *Mmp9*. Over a period of 15 years, no differences in infections or seroconversion against a number of classically tested animal pathogens were observed between sentinel WT and MMP-9^−/−^ mice from the same unique breeding insulator. In addition, microbiome profiling with 16S recombinant DNA (rDNA) sequencing indicated that WT and MMP-9^−/−^ mice had similar microbiota richness and composition in control conditions and after challenge with DSS^12^.

### Similar loss of body weight in MMP-9^−/−^ and WT mice

**Figure 1:**
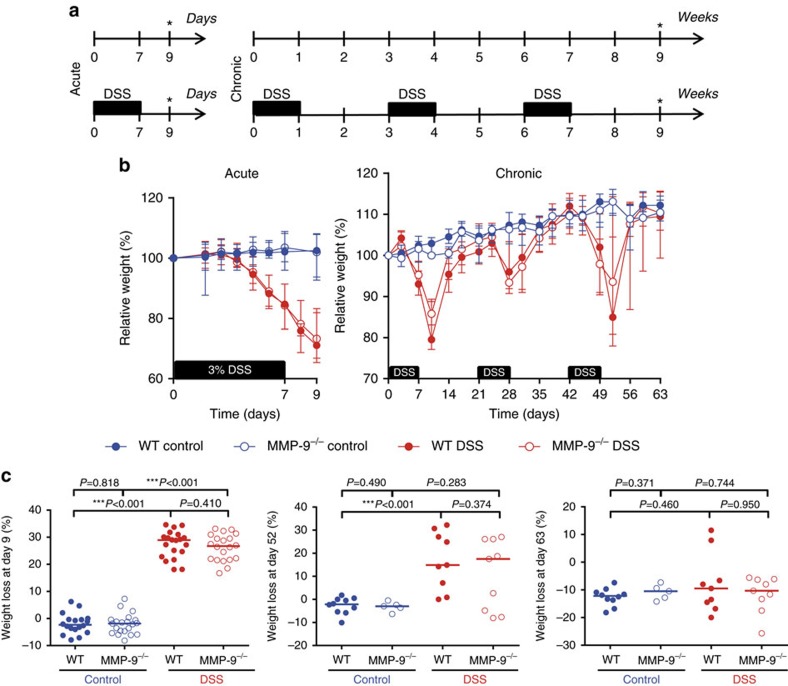
Experimental set-up and body weight analysis after induction of acute and chronic colitis with DSS. (**a**) Acute colitis was induced in male and female WT and MMP-9^−/−^ mice by oral administration of 3% DSS for 7 days in drinking water followed by 2 days of normal drinking water before killing at day 9 (*). Pooled data from three replicated acute DSS experiments are shown. The pooled group of acute DSS exposed mice comprised of WT mice (*n*=20) and MMP-9^−/−^ mice (*n*=20). Control mice received normal drinking water throughout the experiments (WT mice (*n*=18) and MMP-9^−/−^ mice (*n*=21)). To induce chronic colitis, three cycles of 1.75–2.00% DSS were administered, whereby one cycle of DSS comprised 1 week of DSS followed by a recovery period of 2 weeks with normal drinking water. All mice were killed at day 63 (*). Pooled data from three separate chronic DSS experiments are shown. The pooled group of chronic DSS exposed mice comprised WT mice (*n*=9) and MMP-9^−/−^ mice (*n*=9). Control mice received normal drinking water throughout the experiments (WT mice (*n*=10), MMP-9^−/−^ mice (*n*=5)). (**b**) Relative body weight curves of WT and MMP-9^−/−^ mice in acute (left panel) and chronic (right panel) DSS colitis. (**c**) Left panel: absolute body weight loss of mice included in the acute model at day 9. Middle panel: absolute body weight loss of mice included in the chronic model at day 52. Right panel: absolute body weight loss of mice included in the chronic model at day 63. Median values with interquartile range are represented when applicable. Statistical analyses were performed with Mann–Whitney *U* tests (***P≤0.001).

After induction of acute colitis with DSS (Fig. 1a), the relative body weight curves of WT and MMP-9^−/−^ mice changed similarly (Fig. 1b). At the time of killing (day 9), both DSS-treated WT and MMP-9^−/−^ mice had significant loss of body weight compared to corresponding control mice (Fig. 1c). However, no body weight loss differences were observed between DSS-treated WT and MMP-9^−/−^ mice. In addition, we studied chronic long-term effects of MMP-9 gene knockout (KO) in a second model of colitis, recently developed to study intestinal tissue remodelling and fibrosis^13^ (Fig. 1a). After every cycle of DSS, both WT and MMP-9^−/−^ mice lost body weight and subsequently recovered to normal body weights before the start of the next DSS cycle (Fig. 1b). At day 52, 10 days after the third DSS cycle, no significant difference was observed in body weight loss between DSS-treated WT and MMP-9^−/−^ mice. In addition, at the time of killing (day 63), both control and DSS-treated WT and MMP-9^−/−^ mice recovered to normal body weights compared to day 0 (Fig. 1c).

### Similar acute colonic inflammation in MMP-9^−/−^ and WT mice

**Figure 2:**
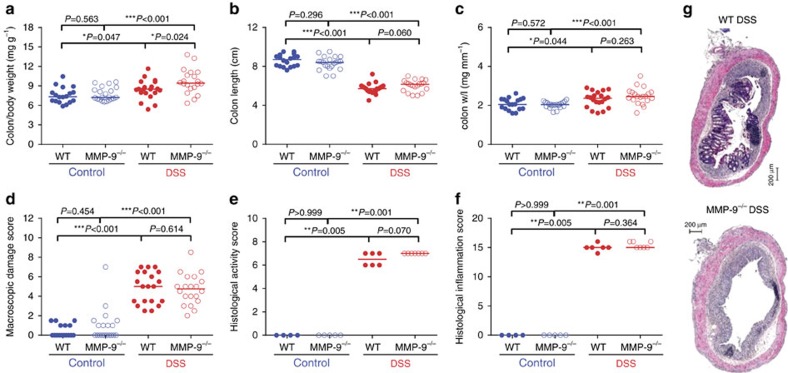
Acute colonic inflammation induced by DSS in MMP-9^−/−^ mice compared to WT mice. Data are shown of acute DSS exposed WT (*n*=20) and MMP-9^−/−^ mice (*n*=20) as well as control WT (*n*=18) and MMP-9^−/−^ mice (*n*=21). Pooled data from three replicated acute DSS experiments are shown. Colon/body weight ratio (**a**), colon length (**b**) colon weight/length (w/l) ratio (**c**) and macroscopic damage score (**d**) were measured in WT and MMP-9^−/−^ mice. Histological activity and inflammation scores (**e**,**f**) were performed on H&E stained colon slides (**g**) in a subset of samples (*n*=24). Median values are represented and statistical analyses were performed with Mann–Whitney *U* tests (*P<0.05, **P≤0.01, ***P≤0.001). Scale bars of 200 μm are shown on the microscopic images.

After acute DSS administration, colon/body weight ratios increased in both WT and MMP-9^−/−^ mice compared to corresponding control mice and were significantly higher in DSS-treated MMP-9^−/−^ mice compared to WT mice (Fig. 2a). Colon length decreased in both DSS-treated WT and MMP-9^−/−^ mice compared to control mice, although no significant difference was found between WT and MMP-9^−/−^ mice after DSS (Fig. 2b). Colon weight/length ratio (Fig. 2c), macroscopic damage score (Fig. 2d) and histological inflammation, and activity scores (Fig. 2e–g) increased after acute administration of DSS, but no differences were found between DSS-treated WT and MMP-9^−/−^ mice. Both male and female mice were used in the acute DSS model. Gender differences were found to be minimal (Supplementary Fig. 2) and were mainly attributed to differences in colon weight. In general, the colon/body weight ratio was significantly lower in male mice compared to female mice, with exception of DSS-treated male MMP-9^−/−^ mice that had a similar colon/body weight compared to female DSS-treated MMP-9^−/−^ mice.

### Similar chronic colonic inflammation in MMP-9^−/−^ and WT mice

**Figure 3:**
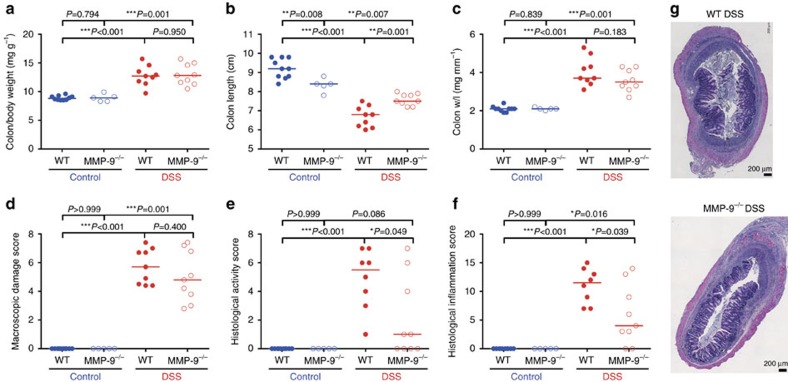
Chronic colonic inflammation induced by DSS in MMP-9^−/−^ mice compared to WT mice. Read-outs from chronic DSS exposed WT (*n*=9) and MMP-9^−/−^ mice (*n*=9), and control WT (*n*=10) and MMP-9^−/−^ mice (*n*=5) are shown. Colon/body weight ratio (**a**), colon length (**b**) colon weight/length (w/l) ratio (**c**) and macroscopic damage score (**d**) were measured in WT and MMP-9^−/−^ mice. Histological activity and inflammation scores (**e**,**f**) were performed on H&E stained colon slides (**g**). Median values are represented and statistical analyses were performed with Mann–Whitney *U* tests (*P<0.05, **P≤0.01, ***P≤0.001). Scale bars of 200 μm are shown on the microscopic images.

After chronic DSS administration, colon/body weight ratios increased in both WT and MMP-9^−/−^ mice compared to control conditions (Fig. 3a). However, no differences were observed in colon/body weight ratios between DSS-treated WT and MMP-9^−/−^ mice (Fig. 3a). Colon length was significantly shorter in MMP-9^−/−^ control mice and longer in DSS-treated MMP-9^−/−^ mice compared to corresponding WT mice (Fig. 3b). Significant increases in colon weight/length ratio (Fig. 3c) and macroscopic damage score (Fig. 3d) were observed after chronic DSS exposure in both WT and MMP-9^−/−^ mice. However, no differences were found between DSS-treated WT and MMP-9^−/−^ mice. Histological inflammation and activity scores were higher in WT mice compared to MMP-9^−/−^ mice after DSS (Fig. 3e–g). This difference was mainly driven by the fact that less epithelial defects and less goblet cell loss were observed in chronic DSS-treated MMP-9^−/−^ mice compared to WT mice (Supplementary Fig. 3d,e).

### Similar remodelling and fibrosis in MMP-9^−/−^ and WT mice

**Figure 4:**
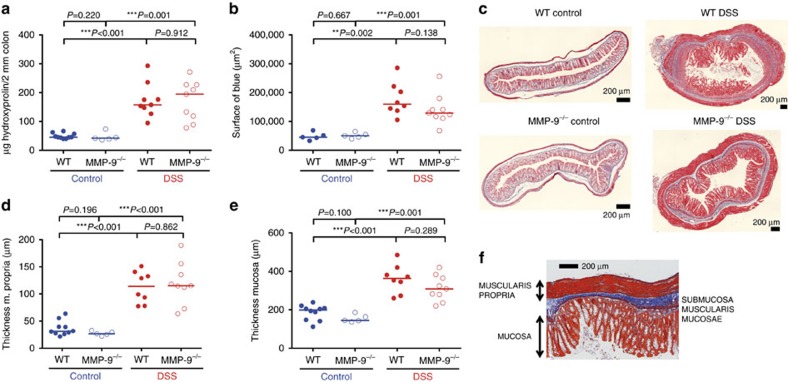
Tissue remodelling and fibrosis parameters after induction of chronic colitis with DSS in WT and MMP-9^−/−^ mice. Read-outs from chronic DSS exposed WT (*n*=9) and MMP-9^−/−^ mice (*n*=9) as well as control WT (*n*=10) and MMP-9^−/−^ mice (*n*=5) are shown. Colonic collagen content was determined using a hydroxyproline assay and quantified as μg hydroxyproline in 2 mm trans sectioned colon (**a**). MSB trichrome staining was performed on colonic tissue sections and the surface area of blue staining was quantified as a measure of collagen deposition (**b**,**c**). The thickness of the muscularis propria (**d**) and the mucosa (**e**) were determined on MSB stained sections (**f**). Median values are represented and statistical analyses were performed with Mann–Whitney *U* tests (**P≤0.01, ***P≤0.001). Scale bars of 200 μm are shown on the microscopic images.

The amount of collagen (Fig. 4a) and the surface area of blue after Martius-Scarlet-Blue (MSB) staining (Fig. 4b,c) significantly increased after chronic DSS administration in both WT and MMP-9^−/−^ mice compared to corresponding control mice. However, these parameters of fibrosis were not significantly different between chronic DSS-treated WT and MMP-9^−/−^ mice. Furthermore, as parameters of tissue remodelling, the thickness of the muscularis propria and the mucosa was not significantly different between chronic DSS-treated WT and MMP-9^−/−^ mice (Fig. 4d–f).

### Similar systemic inflammation in MMP-9^−/−^ and WT mice

Spleen weight, spleen/body weight ratio and spleen/colon weight ratio were not significantly different between WT and MMP-9^−/−^ mice after acute DSS administration (Supplementary Fig. 4a–c). The disease activity index (DAI) increased after acute DSS administration in both WT and MMP-9^−/−^ mice, but no difference was observed between acute DSS-treated WT and MMP-9^−/−^ mice (Supplementary Fig. 4d). After chronic DSS administration, spleen weight and spleen/body weight ratio increased after DSS in both WT and MMP-9^−/−^ mice, although no difference was seen between DSS-treated WT and MMP-9^−/−^ mice (Supplementary Fig. 4e,f). Spleen/colon weight was not significantly altered in MMP-9^−/−^ mice after chronic DSS administration compared to control MMP-9^−/−^ mice (Supplementary Fig. 4g). The DAI was lower in MMP-9^−/−^ mice with chronic DSS colitis compared to WT mice (Supplementary Fig. 4h).

### Similar MMP-2 protein levels in WT and MMP-9^−/−^ mice

**Figure 5:**
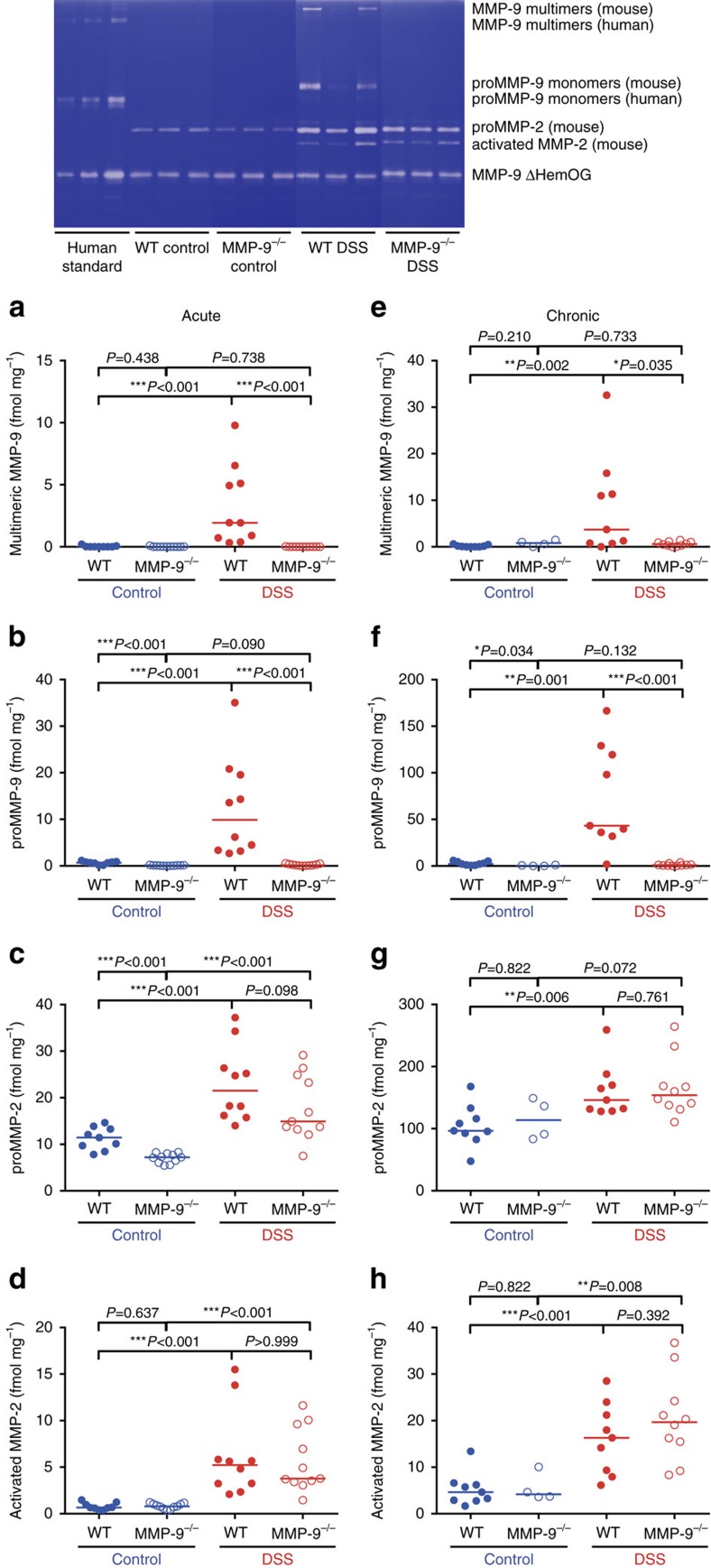
Gelatin zymography analysis of colonic gelatinase A (MMP-2) and gelatinase B (MMP-9) levels in DSS colitis. Data are shown from acute DSS exposed WT (*n*=10) and MMP-9^−/−^ mice (*n*=11) as well as control WT (*n*=9) and MMP-9^−/−^ mice (*n*=11). For the chronic model, read-outs from DSS exposed WT (*n*=9) and MMP-9^−/−^ mice (*n*=10), and control WT (*n*=9) and MMP-9^−/−^ mice (*n*=4) are shown. Pooled data from three replicated acute DSS experiments are shown. A template of a zymography gel (top panel) including samples from control and DSS-treated WT and MMP-9^−/−^ mice is shown. For calibration purposes, three different amounts of three human MMP-9 forms (multimers, monomers and a deletion mutant lacking the haemopexin (Hem) and O-glycosylated (OG) domain) were included. In addition, the latter human deletion mutant protein (MMP-9 ΔHemOG) was spiked into all mouse samples to control sample loading and processing. Quantifications of different MMP-9 and MMP-2 forms in colonic tissue isolated from mice included in the acute model of colitis (**a**–**d**) and the chronic model of colitis (**e**–**h**) are illustrated. Median values are represented and statistical analyses were performed with Mann–Whitney *U* tests (*P<0.05, **P≤0.01 and ***P≤0.001).

As to be expected, we did not observe MMP-9 protein levels in colonic tissue from control or acute and chronic DSS-treated MMP-9^−/−^ mice (Fig. 5a,b,e,f). In contrast, low proMMP-9 levels were detected in colonic tissue from WT control mice (Fig. 5b) and both proMMP-9 trimer and monomer levels significantly increased after acute DSS administration in WT mice (Fig. 5a,b). Serendipitously, lower proMMP-2 levels were found in colonic tissue from control MMP-9^−/−^ mice compared to WT mice (Fig. 5c). After acute DSS administration, proMMP-2 levels increased in both WT and MMP-9^−/−^ mice compared to corresponding control mice (Fig. 5c). In line with these data, at the messenger RNA (mRNA) level (*vide infra*), we observed that *Mmp2* expression was increased in colonic tissue from both MMP-9^−/−^ and WT mice after acute DSS administration. However, no differences in proMMP-2 and activated MMP-2 levels were found between acute DSS-treated WT and MMP-9^−/−^ mice (Fig. 5c,d). After induction of chronic colitis, proMMP-9 trimer and monomer levels were significantly increased in colonic tissue from DSS-treated WT mice compared to control WT mice (Fig. 5e,f). Intriguingly, proMMP-2 levels were significantly increased in WT mice, but not in MMP-9^−/−^ mice after chronic DSS administration (Fig. 5g). Activated MMP-2 levels increased in both WT and MMP-9^−/−^ mice after chronic DSS administration (Fig. 5h).

### Colonic gene expression profiles of WT and MMP-9^−/−^ mice

Since macroscopic and microscopic analyses did not reveal significant differences between DSS-treated WT and MMP-9^−/−^ mice, we sought molecular explanations for the contrasts between the reported phenotypes^6^,^7^,^8^ and the present study. We here report RNA sequencing data of colonic mRNA isolated from WT and MMP-9^−/−^ mice in control and acute DSS conditions. Specifically looking at the alignments of reads to the *Mmp9* gene, several reads mapped (*n*_max_=13) to exons 9–13 (representing the haemopexin domain of MMP-9) in MMP-9^−/−^ control samples, but not to the genetically deleted exons 1–8, whereas in WT control samples reads (*n*<10) were mapped to the full *Mmp9* gene (exons 1–13), as expected^14^. Moreover, in DSS-treated WT mice the amount of reads mapped to exons 1–13 of the *Mmp9* gene was significantly higher than in control WT mice, attesting that *Mmp9* expression is locally increased in the colon by DSS. With these data, we illustrated also maintenance of the original construct in the germ line for 15 years over 13 generations and showed, in contrast with the leaky alternative line^6^,^7^,^8^,^15^, that our mouse line is catalytically dead with a read-through transcript of the *Mmp9* haemopexin domain.

**Figure 6:**
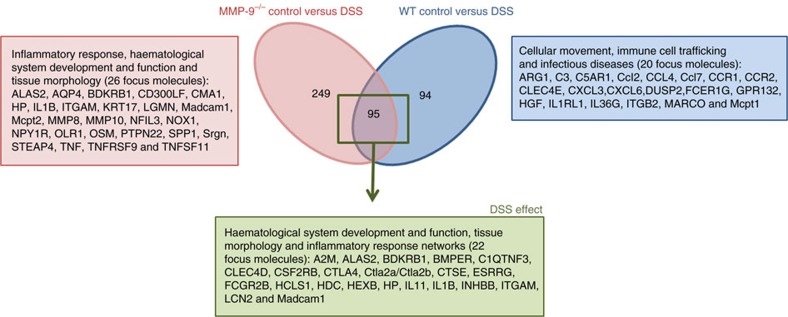
Venn diagram of unique and overlapping DE genes in WT and MMP-9^−/−^ mice after induction of acute colitis with DSS. Unique differentially expressed (DE) genes between control and DSS-induced MMP-9^−/−^ and WT mice are shown in red and blue, respectively. DE genes that overlapped in both comparisons and thus represent the effect of DSS, are shown in green. For both unique and overlapping DE genes, a selection of focus molecules is given based on the top involved pathways (identified with Ingenuity Pathway Analysis).

For differential expression analysis, the overlap between three different methods (DESeq, EdgeR and CuffDiff2) regarding significantly (false discovery rate (FDR) 10% and fold change (FC)>2) differentially expressed (DE) genes was calculated. First, we investigated differences in gene expression between both genotypes. Comparison of gene expression between WT and MMP-9^−/−^ control mice indicated that *Mmp9, Rims4* and *Slpi* were the only three significantly DE genes (Supplementary Table 1). *Rims4* expression was decreased in MMP-9^−/−^ mice, whereas the expression of *Slpi* was increased in MMP-9^−/−^ mice, compared to WT mice. After induction of acute colitis with DSS, we found with EdgeR and CuffDiff2 analyses that the expression of 11 genes (*Clps, Ddx60, Fgb, Fgg, Ifi44, Ifit2, Isg15, Itih3, Itih4, Oas3* and *Usp18*), which are involved in antimicrobial response, was increased in MMP-9^−/−^ compared to WT mice (Supplementary Table 1). Second, we investigated the effect of MMP-9 gene KO regardless of the fact whether mice received DSS. Therefore, we looked at the overlapping DE genes between control and DSS-induced WT *versus* MMP-9^−/−^ mice. We found no overlap in DE genes between these comparisons with DESeq or EdgeR, whereas with CuffDiff2 we found 1 overlapping DE gene (pre B-lymphocyte 3/*Vpreb3)* of which the expression was decreased in MMP-9^−/−^ mice compared to WT mice regardless of the induction of inflammation. Third, we studied the effect of DSS in both genotypes separately. We found that 189 annotated DE genes were induced by DSS in WT mice and overlapped between the three differential expression analysis methods (Fig. 6). In the MMP-9^−/−^ mice, we annotated 344 DSS-induced DE genes that overlapped between the three differential expression analysis methods (Fig. 6). Finally, we found 95 annotated DE genes that were common to both WT and MMP-9^−/−^ mice in response to an inflammatory stimulus and these genes were involved mainly in haematological system development and function, tissue morphology and inflammatory response networks (Fig. 6). In contrast, 94 and 249 annotated DE genes were specific to WT and MMP-9^−/−^ mice, respectively, in their response to inflammation (Fig. 6). Pathway analysis indicated that the 94 genes that were uniquely DE in WT mice after DSS were involved in cellular movement, immune cell trafficking and infectious diseases networks (Fig. 6). The 249 genes that were uniquely DE in MMP-9^−/−^ mice after DSS were involved in inflammatory response, haematological system development and function and tissue morphology networks (Fig. 6).

In addition, we specified differential expression of other *Mmp* or *Timp* genes. We found that there was no significantly different expression of *Mmp* or *Timp* genes between WT and MMP-9^−/−^ mice under control or DSS conditions (with exception of *Mmp9*; Table 1). Interestingly, when looking at the response to DSS in WT and MMP-9^−/−^ separately, we found that in WT mice the expression of *Mmp3, Mmp8, Mmp9*, *Mmp10, Mmp12*, *Mmp13, Mmp19* and *Timp1* was increased after DSS, whereas in MMP-9^−/−^ mice only *Mmp8* and *Mmp10* were significantly upregulated after DSS (Table 1).

### Similar TNBS-induced colitis in MMP-9^−/−^ and WT mice

**Figure 7:**
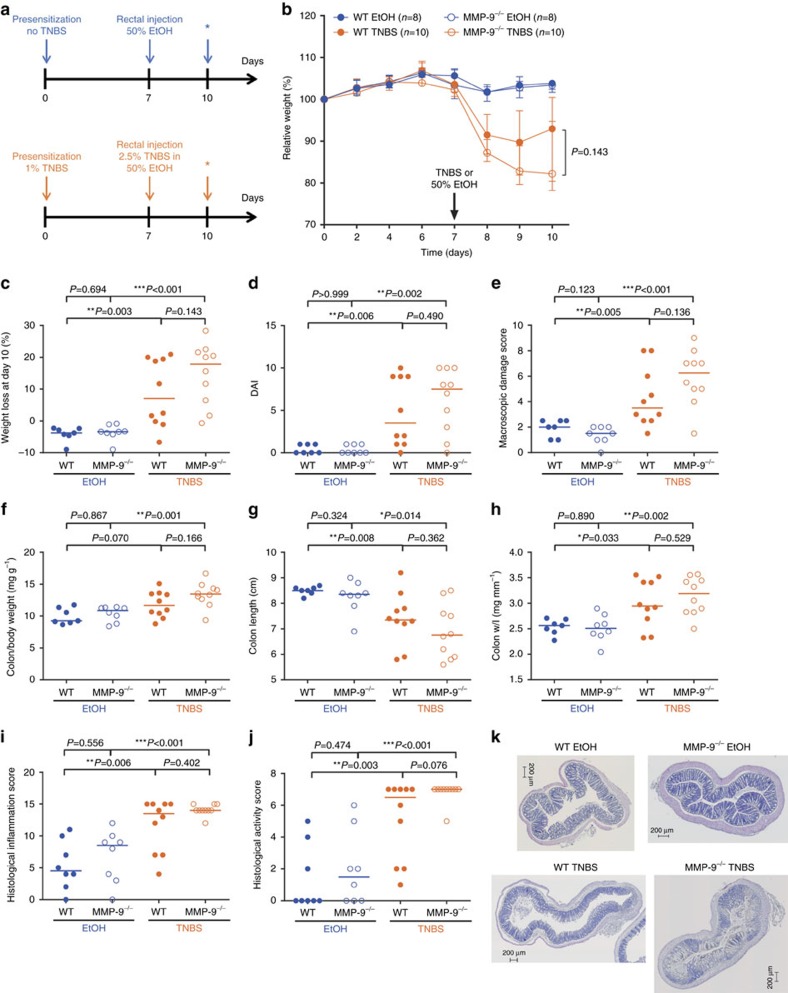
Parameters of inflammation after induction of acute colitis with TNBS in WT and MMP-9^−/−^ mice. Data are shown from TNBS exposed WT (*n*=10) and MMP-9^−/−^ (*n*=10) mice as well as control WT (*n*=8) and MMP-9^−/−^ (*n*=8) mice. The experimental set-up for control and TNBS-treated mice included presensitization with or without TNBS at day 0, followed by rectal injection of either 50% EtOH or 2.5% TNBS in 50% EtOH at day 7 and killing of all mice at day 10 (*) (**a**). As clinical parameters, relative weight curves (**b**), absolute weight loss at time of killing (**c**) and DAI (**d**) are shown. The macroscopic damage score (**e**), colon/body weight ratio (**f**), colon length (**g**) and colon weight/length (w/l) ratio (**h**) are shown as parameters of macroscopic colonic inflammation. Finally, histological inflammation (**i**) and histological active disease scores (**j**) are shown, in addition with H&E illustrations (**k**) of pieces of colon from EtOH- and TNBS-induced WT and MMP-9^−/−^ mice. Median values with interquartile range are represented when applicable. Statistical analyses were performed with Mann–Whitney *U* tests (*P<0.05, **P≤0.01 and ***P≤0.001). Scale bars of 200 μm are shown on the microscopic images.

To confirm the lack of difference in phenotype between WT and MMP-9^−/−^ mice after induction of colitis with DSS, which more closely resembles UC, we performed a TNBS colitis model (Fig. 7a) which has a different immunological mechanism and is more related to CD in humans^3^. We found that both WT and MMP-9^−/−^ mice lost significant amounts of body weight after rectal administration of TNBS compared to corresponding control mice that received 50% EtOH (Fig. 7b). However, there was no significant difference in body weight loss between TNBS-treated WT and MMP-9^−/−^ mice at time of killing (Fig. 7c). In addition, DAI, macroscopic damage score and colonic measurements also increased after TNBS in both WT and MMP-9^−/−^ mice compared to corresponding controls, but we observed no significant differences between TNBS-treated WT and MMP-9^−/−^ mice (Fig. 7d–h). Histological evaluation indicated that the histological inflammation and activity scores were similar between control WT and MMP-9^−/−^ mice that were rectally injected with 50% EtOH (Fig. 7i–k). A significant increase in histological inflammation and activity scores was observed in TNBS-induced mice compared to 50% EtOH-induced mice (Fig. 7i–k). However, similar histological inflammation and activity scores were observed in TNBS-induced WT and MMP-9^−/−^ mice (Fig. 7i–k).

### Pharmacological inhibition of MMP-9 does not improve colitis

**Figure 8:**
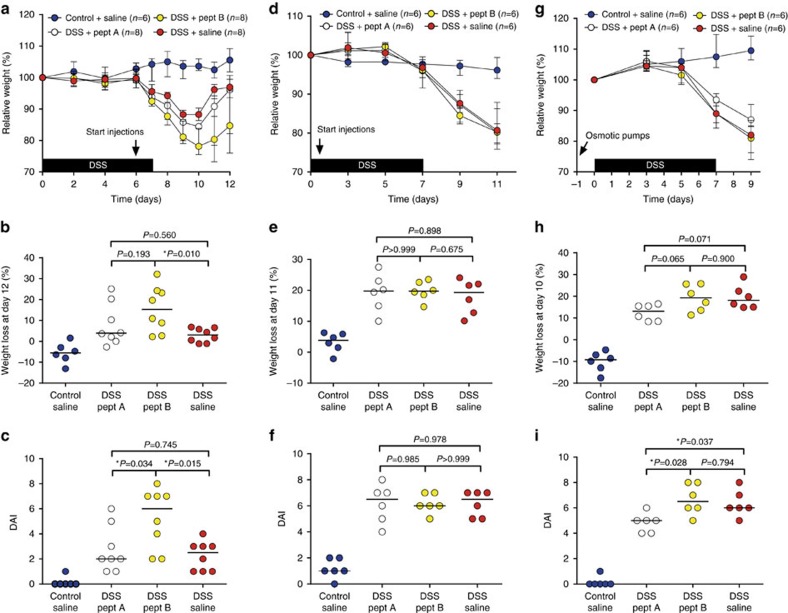
Body weight loss and DAI of C57BL/6J mice with acute DSS-induced colitis treated with peptide inhibitors. Representation of relative body weight loss, absolute body weight loss at time of killing and DAI for control and DSS-induced mice included in the therapeutic scheme (*n*=30) (**a**–**c**), the prophylactic scheme (*n*=24) (**d**–**f**) and for control and DSS-induced mice subcutaneously implanted with osmotic pumps yielding continuous delivery of sufficient amounts of peptide inhibitor (*n*=24) (**g**–**i**). Median values with interquartile range are represented when applicable. Statistical analyses were performed with Mann–Whitney *U* tests (*P<0.05).

**Figure 9:**
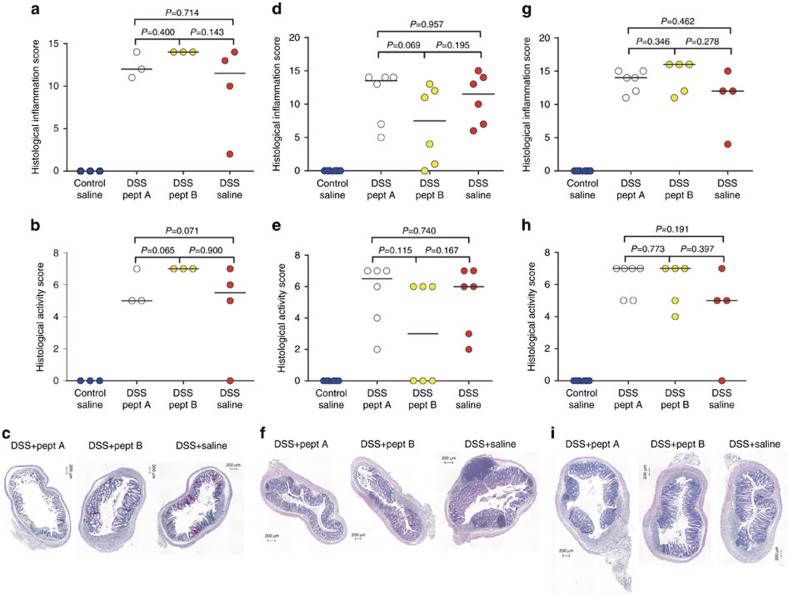
Histological inflammation and activity scores after peptide inhibitor administration. Histological scores are shown after therapeutic administration (*n*=13) (**a**–**c**), prophylactic administration (*n*=24) (**d**–**f**) and osmotic pump delivery of peptide inhibitors (*n*=21) (**g**–**i**), respectively. Median values are represented and statistical analyses were performed with Mann–Whitney *U* tests. Scale bars of 200 μm are shown on the microscopic images.

First, intraperitoneal (i.p.) injections of peptide inhibitors A and B (refs 16, 17) were given daily to DSS-treated mice starting from day 6 (after development of colitis, therapeutic scheme; Fig. 8a). Control mice were injected daily with 0.9% pyrogen-free NaCl (saline). DSS-treated mice injected with peptide inhibitor B had significantly more body weight loss at time of killing compared to DSS-treated mice injected with saline (Fig. 8b). However, no difference in body weight loss was observed in DSS-induced mice treated with peptide inhibitor A compared to peptide inhibitor B or saline (Fig. 8b). Moreover, the DAI of DSS-induced mice that were injected with peptide inhibitor B was higher compared to DSS-induced mice injected with either peptide inhibitor A or saline, although no difference was observed between peptide inhibitor A injected DSS-induced mice and saline injected DSS-induced mice (Fig. 8c). Second, DSS-treated mice were injected daily with peptide inhibitor A or B starting from day 1 (before development of colitis, prophylactic scheme; Fig. 8d). DSS-treated mice lost significantly more body weight, compared to control mice, although no differences were observed between DSS-treated mice receiving peptide inhibitor A, peptide inhibitor B or saline (Fig. 8e). Moreover, the DAI was not significantly different between saline, peptide inhibitor A or peptide inhibitor B injected DSS-induced mice (Fig. 8f). Finally, since the half-life of these peptide inhibitors in the circulation is quite low (<1 h)^16^, we implanted osmotic pumps subcutaneously on the back of the mice ensuring a continuous release of the inhibitors. All mice lost significant amounts of body weight after DSS (Fig. 8g). However, administration of peptide inhibitor A resulted in a mild protection with lower loss of body weight in DSS-treated mice compared to peptide inhibitor B and saline (Fig. 8h). This was also reflected by the DAI, whereby DSS-treated mice had a significantly lower DAI after delivery of peptide inhibitor A compared to peptide inhibitor B or saline (Fig. 8i). By histopathological analysis, we did not observe a significant effect of peptide inhibitor A nor B on the severity of intestinal inflammation after all three administration schemes (Fig. 9).

The macroscopic damage score of DSS-treated mice was not altered by peptide inhibitors A or B in the therapeutic (Supplementary Fig. 5a,b) or prophylactic schemes (Supplementary Fig. 6a,b). However, when peptide inhibitors were continuously administered via osmotic pumps, a lower macroscopic damage score was observed in DSS-induced mice treated with peptide inhibitor A compared to DSS-induced mice that received saline (Supplementary Fig. 7a,b). Colonic measurements were not significantly different in DSS-treated mice that received peptide inhibitors compared to saline injection in a therapeutic (Supplementary Fig. 5) or prophylactic (Supplementary Fig. 6) scheme. However, after continuous release of the peptide inhibitors through osmotic pump delivery, the colon length of DSS-treated mice that received peptide inhibitor A was significantly longer compared to the colon length of DSS-treated mice that were given peptide inhibitor B or saline (Supplementary Fig. 7c). However, other colonic measurements (colon weight, colon/body weight ratio and colon weight/length ratio) were not significantly different between DSS-induced mice that received peptide inhibitor A, peptide inhibitor B or saline through osmotic pump delivery (Supplementary Fig. 7d–f). To ensure that therapeutic levels of peptide inhibitors A and B were attained during all three administration schemes, plasma levels were measured with competitive ELISAs as described previously^16^. As shown in supplementary Fig. 8, inhibitory plasma levels were indeed reached *in vivo* for all three administration schemes.

### MMP inhibition alters colonic MMP mRNA expression

**Figure 10:**
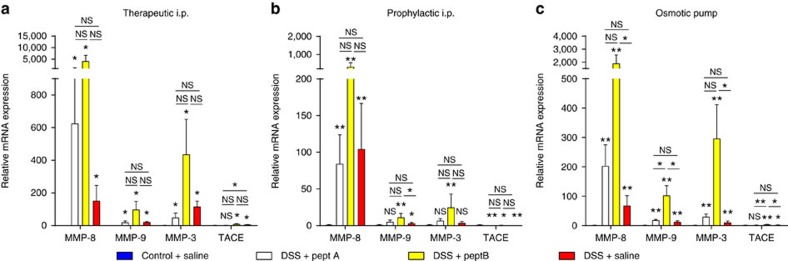
Relative mRNA expression of specific *Mmps* and *Tace* after administration of peptide MMP inhibitors. Relative mRNA expression measured in colonic mouse tissue after a therapeutic injection scheme (*n*=16) (**a**), a prophylactic injection scheme (*n*=23) (**b**) and continuous osmotic pump delivery (*n*=22) (**c**) of peptide inhibitors A and B. Expression levels were normalized to the control condition (water+saline) and 18S with the use of the 2-delta delta CT method^48^. Mean values with s.e.m. are shown. Statistical analyses were performed with Mann–Whitney *U* tests (*P<0.05, **P≤0.01; NS, not significant). Colour codes are similar as in Figs 8 and 9.

In view of the known effects of peptide inhibitors A and B on MMP-3, MMP-8, MMP-9 and TACE (refs 16, 17), on the basis that MMP-3 is an activator of MMP-9 (ref. 18) and because MMP-8 and MMP-9 are major neutrophil MMPs (refs 3, 4, 5), we evaluated colonic expression levels of specific mRNAs in control and DSS-induced and peptide-treated mice. As expected, mice with acute DSS-induced colitis had significantly increased expression of *Mmp3*, *Mmp8* and *Mmp9* compared to control mice (Fig. 10a–c). In the therapeutic scheme, the expression of *Mmp3, Mmp8* and *Mmp9* appeared to be higher in DSS-treated mice injected with peptide inhibitor B compared to peptide inhibitor A or saline (Fig. 10a). In the prophylactic scheme, the same trend was observed and, in addition, DSS-treated mice injected with peptide inhibitor B had a significantly increased expression of *Mmp9* compared to DSS-treated mice injected with saline (Fig. 10b). When peptide inhibitors were delivered continuously via implanted osmotic pumps, *Mmp8* and *Mmp3* expression was significantly higher in DSS-induced mice that received peptide inhibitor B compared to saline (Fig. 10c). Moreover, *Mmp9* expression was significantly increased in DSS-induced mice that received peptide inhibitor B compared with peptide inhibitor A or saline (Fig. 10c). Significant differences in *Tace* expression were seen after all three administration schemes, although relative expression values were low in comparison with those of the analysed MMPs. These data attest that with the three treatment schemes, in addition to reaching inhibitory plasma levels (Supplementary Fig. 8), pharmacological effects were also locally demonstrated in colonic mucosa.

## Discussion

Various studies point towards a disease causing role of MMP-9 in animal models of IBD^6^,^7^,^8^,^9^,^10^,^19^. We here critically re-evaluated these studies and reduced possible confounding parameters of gene KO studies. We optimized control settings by using sufficiently large animal cohorts, by performing sufficient backcrosses and by using the same environmental conditions for breeding WT and MMP-9^−/−^ mice for more than 15 years. In addition, SNP analysis and RNA sequencing were performed to detect differences at the DNA and mRNA levels, and gelatin zymography analysis was used to show absence of MMP-9-mediated gelatinolysis after faithfully introducing the KO construct *in vivo*. Finally, 16S rDNA sequencing was performed to study differences in microbiota richness and composition between WT and MMP-9^−/−^ mice in control and DSS conditions^12^. Briefly, we observed that changes in gut microbiota were mainly driven by DSS and were not significantly altered by MMP-9 gene KO.

First, potential differences between WT and MMP-9^−/−^ mice after induction of acute colitis were studied. Therefore, we used two models of chemically induced colitis, namely DSS and TNBS, that are driven by different immunological mechanisms^3^. The DSS model more closely resembles UC, whereas TNBS is more representative for CD in humans. Surprisingly, our genetically well-defined MMP-9^−/−^ mice did not have the reported^6^,^7^,^8^ attenuation of colitis phenotypes after DSS or TNBS administration compared to WT mice. The colon/body weight, however, was found to be significantly higher in MMP-9^−/−^ mice after DSS administration, but this was related to a gender-driven effect with higher colon/body weight in male mice. Second, since IBD is characterized by a chronic disease course with tissue remodelling and fibrosis as important complications, we additionally studied the effect of MMP-9 gene KO on chronic DSS-induced colitis and fibrosis. However, the majority of the inflammatory, tissue remodelling and fibrosis parameters were not significantly different between MMP-9^−/−^ and WT mice after chronic DSS administration. In conclusion, we did not observe a causal role of MMP-9 in three different models of colitis: two acute inflammatory models and one chronic fibrostenosing model.

These discrepancies in study outcomes can be explained at several levels. First, it needs to be remarked that our study cohorts were larger than those presented in other studies^6^,^7^,^8^. Studies with small cohorts have the intrinsic risk of larger bias. Second, our WT and MMP-9^−/−^ mice were kept for more than 15 years in the same SPF insulator with exactly the same environmental conditions and this aspect was not considered in previous studies^6^,^7^,^8^. Third, because of a subfertility phenotype it took more than 10 years to obtain black MMP-9^−/−^ mice in our backcross experiments into C57BL/6J background (Supplementary Fig. 1). Although the importance of sufficient backcrosses is widely recognized^20^, it remains arbitrary. However, mice with different fur colour not only have different genetic backgrounds, they also may cause observation bias. In other IBD/MMP-9 studies^6^,^7^,^8^, genetic background and for example, fur colour are not detailed. In the present study, we used black MMP-9^−/−^ mice from the 13th backcross into C57BL/6J (Supplementary Fig. 1) and showed absence of a functional *Mmp9* gene in our in-house bred MMP-9^−/−^ mice at the DNA, mRNA and protein levels. Genetic background characterization with 1,449 SNPs indicated that our KO mice are 99.86% on C57BL/6J background and are matched to the genetic background of their WT littermates, with exception of 2 SNPs on the 129S6/Sv background which are related to the region of genetic modification for *Mmp9* (ref. 14). By RNA sequencing of colonic tissues, we documented that in the processed mRNA, exons 1–8 were indeed absent. In addition, we observed an artificial read-through transcript, that is, the haemopexin domain (exons 9–13). This exemplifies that more attention than previously thought needs to be paid to control experiments in all KO studies. At the protein level, we confirmed the absence of a functional MMP-9 enzyme in our MMP-9^−/−^ mice with gelatin zymographies^14^ (Fig. 5). In contrast, in the previously published IBD/MMP-9 studies^6^,^7^,^8^, a mouse strain with demonstrated functional leakiness at the MMP-9 protein level^15^ was used. Whereas some evidence for the leakiness of MMP-9 expression in the previously used IBD/MMP-9^−/−^ mice is present in two published manuscripts^6^,^8^, no zymography analysis was provided in a third study^7^.

Since we did not observe significant macroscopic and histopathological differences in the absence of MMP-9 in a setting of acute intestinal inflammation that was similar to the ones reported previously^6^,^7^,^8^, we performed in-depth analysis with whole-genome RNA sequencing. We found that only 3 genes (*Mmp9, Rims4* and *Slpi)* were DE in the colon between MMP-9^−/−^ and WT control mice. *Rims4* (regulating synaptic membrane exocytosis 4) expression was found to be sixfold lower in MMP-9^−/−^ mice. Rims4 protein is involved in ion channel binding^21^. Secretory leucocyte peptidase inhibitor (*Slpi*) was found to be 2.5-fold higher in MMP-9^−/−^ mice. It inhibits endopeptidases and microbes and was previously studied in experimental colitis and IBD^22^,^23^,^24^,^25^. Moreover, a more direct link with MMP-9 was found whereby SLPI was shown to promote the metastasis of gastric cancer cells by increasing MMP-9 expression^26^. Interestingly, both *Rims4* and *Slpi* are located on mouse chromosome 2 not far (<1 Mb) from *Mmp9.* After stimulation with DSS, 11 genes (*Clps, Ddx60, Fgb, Fgg, Ifi44, Ifit2, Isg15, Itih3, Itih4, Oas3* and *Usp18*) involved in antimicrobial response were more highly expressed in MMP-9^−/−^ *versus* WT mice. Remarkably, the expression of a number of genes in the interferon response was increased and interferon-β has been described as a substrate of MMP-9 (ref. 27). This finding is reminiscent of increased expression of other MMP-9 substrates in the pancreas of MMP-9^−/−^ mice^28^. Moreover, ISG15 ubiquitin-like modifier (*Isg15*)^29^, fibrinogen beta (*Fgb*) and fibrinogen gamma (*Fgg*) chains^30^ were also identified as substrates of MMP-9. The expression of *Vpreb3/*pre B-lymphocyte protein 3 was found to be decreased in MMP-9^−/−^ mice compared to WT mice in both control and DSS conditions. *Vpreb3* is expressed during B-cell differentiation in subsets of mature B lymphocytes and is linked to formation of B-cell lymphomas^31^. Many genes (*n*=249) were found to be uniquely expressed by MMP-9^−/−^ mice after DSS administration. Interestingly, 25% of these genes were involved in differential regulation of cytokine production in intestinal epithelial cells by IL-17A and IL-17F. This attests to the role of MMP-9 as a tuner of immune functions^32^. In addition, protein tyrosine phosphatase non-receptor 22 (PTPN22) was one of the top molecules uniquely expressed in MMP-9^−/−^ mice after DSS administration. PTPN22 is known to dephosphorylate NLRP3 upon inflammasome induction, allowing efficient NLRP3 activation and subsequent IL-1β release. In murine models, PTPN22 deficiency results in pronounced colitis, increased NLRP3 phosphorylation, but reduced levels of mature IL-1β. Conversely, IBD patients who carry an autoimmunity-associated PTPN22 variant have increased IL-1β levels. PTPN22 is therefore recognized as an important regulator of NLRP3 that prevents aberrant inflamasome activation^33^. The expression of cytotoxic T-lymphocyte-associated protein 4 was upregulated in both WT and MMP-9^−/−^ mice after DSS. CTLA-4 is a coinhibitory protein required for regulation of T-cell activation and CTLA-4 deficiency in mice is associated with fatal lymphoproliferation, intestinal inflammation and autoimmunity. Moreover, neutralization of CTLA-4 is associated with intestinal inflammation and autoimmunity in human cancer. Recently, genetic variants in cytotoxic T-lymphocyte-associated protein 4 were also associated with early-onset CD (ref. 34). In a subset of MMP and TIMP genes, we found that upregulation of *Mmp8* and *Mmp10* by DSS was shared between both genotypes, whereas *Mmp3*, *Mmp9*, *Mmp12, Mmp13, Mmp19* and *Timp1* were uniquely upregulated in WT mice. The general picture emerging from this extensive gene expression analysis is also in line with our microarray data of IBD patients under anti-tumour necrosis factor treatment^35^ and points towards the idea that the disease process induces altered expression of several MMPs and TIMPs^3^, rather than being the result of MMP-9 specifically.

In view of promising effects of monoclonal antibodies against MMP-9 on DSS-induced colitis^9^,^10^ and small peptide inhibitors of MMP-9 on acute endotoxin shock in mice^16^, we expected that treatment with MMP-9 inhibiting peptides would lead to beneficial effects. We therefore treated mice with bolus injections or with a continuous infusion of peptide inhibitors, but observed no overall significantly beneficial effects. In fact, in a therapeutic administration scheme, a trend for an opposite effect was seen for peptide inhibitor B. In contrast, DSS-treated mice that were implanted with osmotic pumps and continuously received peptide inhibitor A did show improved disease activity scores. However, in all three treatment schemes, histological inflammation and disease activity scores were not significantly altered by peptide inhibitor A or B administration compared to saline. Furthermore, mRNA expression of *Mmp3, Mmp8* and *Mmp9* was highly increased in DSS-treated mice that received peptide inhibitor B compared to peptide inhibitor A or saline, with highest upregulation after the therapeutic injection scheme and delivery via implanted osmotic pumps. In general, in line with our data obtained in MMP-9^−/−^ mice, pharmacological inhibition of MMP-9 did not improve colitis phenotypes. Why inhibition of MMP-9 with monoclonal antibodies improves DSS-induced colitis^9^,^10^, whereas inhibition with small peptides does not, is puzzling. To rule out that insufficient levels were attained, we measured the levels of peptide inhibitors A and B in plasma for all three administration schemes as described previously^16^ and confirmed that adequate plasma levels were indeed present at time of killing (Supplementary Fig. 8). Furthermore, local mucosal alterations of MMP and TACE mRNAs were a further illustration of the biological effects of the used peptide inhibitors.

In conclusion our data (i) illustrate that mouse KO experiments need to be better controlled than published in most manuscripts up-to-now and that careful analyses of genetic background, of eventual leakiness at the mRNA and protein levels and of different environmental factors between used WT and KO mice need to be detailed, (ii) place important question marks behind the previously reported causal role of MMP-9 in murine colitis and (iii) call for scrutiny in preclinical and clinical tests of prophylactic and therapeutic MMP inhibition for IBD. However, these data (iv) reinforce in murine animal studies that MMP-9 constitutes an excellent inflammatory marker for IBD.

## Methods

### Mice

Initially, we developed two MMP-9^−/−^ mouse lines at the Rega Institute for Medical Research (Leuven, Belgium), one of which turned out to still produce MMP-9 and the other one not. The former leaky line was immunologically tolerant for mouse MMP-9, whereas in the latter non-leaky mouse line we developed neutralizing antibodies against mouse MMP-9 (refs 14, 36). However, our catalytically dead mouse line had a subfertility phenotype with low pup numbers and this considerably slowed down our backcrossing scheme^37^. After about 10 years of backcrossing into the C57BL/6J background, we were at generation 10, but these mice had still agouti fur colour^38^. Finally, by several additional years of backcrossing, we obtained black MMP-9^−/−^ mice in the 13th generation (Supplementary Fig. 1). All mice used in this study were confirmed for their MMP-9^−/−^ or WT genotype by PCR. In addition to KO status, we carefully characterized the genetic background of the mice. Genomic DNA was extracted from tail tips of the mice and background strain characterization was performed on a panel of 1,449 SNP markers (Taconic, Rensselaer, NY, USA), as detailed elsewhere^39^. MMP-9^−/−^ mice and their WT littermates were reared under SPF conditions at the Rega Institute for Medical Research (Leuven, Belgium) for more than 15 years within the same insulator with access to the same formula of nutrition and thereby with unprecedented environmental similarities. On a regular basis sentinel WT and MMP-9^−/−^ mice of the same breeding insulator were screened for infections and seroconversion against a number of classical animal pathogens. Moreover, 16S rDNA sequencing was performed to study differences in microbiota richness and composition between WT and MMP-9^−/−^ mice in control and DSS conditions^12^. The study was approved by the local ethics committee for animal experimentation of the University of Leuven (P134–2010, P178–2011 and P156/2016).

### Dextran sodium sulphate-induced colitis

Acute and chronic dextran sodium sulphate (DSS) colitis were induced as described previously^13^,^39^. Acute colitis was induced in 8–10 weeks old male and female mice by oral administration of 2–3% DSS (35–50 kDa; MP Biomedicals, Illkirch, France) in the drinking water during 7 days followed by 2–3 days on normal drinking water. All mice were killed at day 9 (Fig. 1a). Chronic colitis was induced by 3 cycles of DSS in 8–10 weeks old female mice. One cycle of DSS was defined as 1 week of DSS administration followed by a recovery period of 2 weeks on normal drinking water. All mice were killed after 9 weeks (Fig. 1a). Control mice (WT and MMP-9^−/−^) received normal drinking water throughout the duration of the experiment. The body weight of the mice was monitored daily or every other day in case of the acute model, whereas in the chronic model, body weight was followed every 3–4 days. Of importance, WT and MMP-9^−/−^ mice were co-housed (maximally 5 mice per cage) during the experiments ensuring identical environmental conditions as well as controlling for cage-related effects (for example, coprophagia and intake of food and (DSS-supplemented) drinking water). Both the acute and chronic DSS experiments were repeated three times whereby results could be replicated each time and in total 79 mice were used in the acute models and 35 mice in the chronic models. The number of mice needed was estimated based on previous experience^13^ and included at least 5 mice per group to ensure adequate power of detection. In total, 1 MMP-9^−/−^ and 3 WT mice died during the 3 acute DSS experiments, whereas in the 3 chronic models, 1 MMP-9^−/−^ and 1 WT mice died after the third cycle of DSS due to severe weight loss. These mice were excluded from further analyses. In addition, 1 MMP-9^−/−^ mouse was excluded afterwards for the total analysis of the experiment because of severe dysplasia (neoplasia) at the histological level. No randomization was applied before start of the experiments.

### Trinitrobenzenesulfonic acid-induced colitis

Acute 2,4,6-trinitrobenzenesulfonic acid (TNBS) colitis was induced in 7–8 weeks old male and female WT and MMP-9^−/−^ mice as described earlier (Fig. 7a)^40^,^41^. Briefly, 7 days before rectal administration, WT and MMP-9^−/−^ mice were cutaneously presensitized with 1% (w/v) TNBS in a 4:1 volume ratio of aceton and olive oil. Therefore, a small patch of abdominal skin was shaved with an electric razor where after 150 μl of presensitization solution was applied. Control mice were treated with presensitization solution without TNBS. At day 7, mice were anaesthetized by i.p. injection of Ketamine/Xylazin solution. A plastic feeding tube (FTP-20–38, Phymep, France) was fitted to a 1 ml syringe and filled with 50% ethanol (EtOH) or 1:1 volume of 5% TNBS in absolute EtOH (2.5% TNBS). The tube was then gently inserted into the colon 3 cm proximal to the anus and 100 μl of either solution was slowly administered into the lumen. Thereafter, the mice were kept in a vertical position for 60 s and returned to the cage. The body weight of the mice was monitored every other day after presensitization and every day after rectal EtOH or TNBS administration (day 7) until time of killing. In total, 38 mice were included of which 1 MMP-9^−/−^ and 1 WT mice were excluded due to death after either sedation or injection of TNBS, respectively. WT and MMP-9^−/−^ mice were co-housed during the experiment.

### Evaluation of colonic inflammation and histology

Animals were euthanized with sodium pentobarbital (Nembutal, Ovation Pharmaceuticals Inc. Deerfield, USA). The DAI was calculated based on three parameters: loss of body weight (one point for each 5% loss of body weight), consistency of stools (normal=0, soft=2, liquid=4) and presence of gross blood in stools (0=none, 1=present)^13^,^39^. The colon was isolated, weighted and its length measured from the ileocecal junction to the anus. Since our non-leaky MMP-9^−/−^ mice are smaller than their WT littermates^38^, we here report measurements of colon weight as a ratio over body weight. The macroscopic damage score was calculated based on extent of inflammation along the colon (in cm, multiplied by 2 if severe), colonic mesenterial adhesion (0=none, 1=mild, 2=severe) and colonic hyperaemia (0=none and 1=present)^13^,^39^. A piece of colon was fixed in 4% formalin for histopathological evaluation and other parts were snap-frozen for RNA sequencing, quantitative real-time polymerase chain reaction, gelatin zymography and hydroxyproline assay. Histopathological evaluation was performed on paraffin embedded, 5 μm-thick longitudinal and transverse sections stained with haematoxylin and eosin (H&E). The histological inflammation score was calculated based on the sum of architectural changes, neutrophil infiltration, epithelial defects, mononuclear cell infiltration and goblet cell loss; whereas the histological activity score was calculated based on neutrophil infiltration and epithelial defects^13^,^39^. Three sections per animal were evaluated and slides were scored by experienced pathologists (K.G. and G.D.H.) who were blinded to the experimental conditions.

### Evaluation of tissue remodelling and fibrosis

Paraffin embedded, 5 μm-thick transverse sections were stained using a MSB trichrome staining highlighting connective tissue changes^13^,^39^. Images were acquired with the use of a Zeiss Axiovert 200 microscope, a Zeiss Axiocam MRc5 camera and the Zeiss Axiovision 4.7.1.0 software imaging system. As parameters of tissue remodelling, the thickness of the mucosa and muscularis propria were calculated as mean values of two different points per mouse on uniform horizontal cross sections of colon crypts using ImageJ (ref. 42). To evaluate fibrosis, a hydroxyproline assay^43^ was performed to evaluate collagen content in the colon. In addition, the surface of blue (μm^2^) in mucosa and submucosa was quantified on MSB stained slides using ImageJ 1.45 (NIH Windows version)^42^.

### Gelatin zymography

Gelatin zymography was performed in accordance with recent recommendations and standardizations^4^,^35^,^39^,^44^. Snap-frozen colonic tissues from WT and MMP-9^−/−^ mice after both acute and chronic colitis models were prepurified using gelatin Sepharose beads (GE Healthcare, Buckinghamshire, United Kingdom) and mini spin columns (Bio-Rad Laboratories, Hercules, CA, USA)^45^. The bound gelatinases were eluted from the column with 20 μl Tris/glycine/sodium dodecyl sulfate nonreducing loading buffer (Invitrogen, Carlsbad, CA, USA). The prepurified samples were then spiked with a known amount of a recombinant deletion mutant of human MMP-9 that does not interfere with any mouse gelatinolytic enzyme. The spiking reference was included for quantitative standardization. In addition, to identify and qualitatively distinguish between the various forms of mouse MMPs, a standard of human gelatinase B multimers, monomers and a deletion mutant was included on the analytical zymography gels. In this way the monomeric and multimeric forms, the proforms and activation forms of both mouse MMP-2 and MMP-9 were distinguished. The samples were then separated in 7.5% acrylamide gels copolymerized with 1 mg ml^−1^ porcine gelatin (Sigma-Aldrich, St Louis, MO, USA), whereafter the gels were washed with 2.5% Triton X-100 (Sigma-Aldrich) for 40 min and incubated overnight at 37 °C in 50 mM Tris-HCl (pH 7.5) supplemented with 10 mM CaCl_2_ (Sigma-Aldrich). The gels were stained with 0.25% Coomassie Brilliant Blue-R (Sigma-Aldrich) and scanned using standard settings. Band densities were analysed and quantified with ImageJ 1.48 software (NIH Windows version)^42^. Information about weight and protein concentration of all colonic samples was collected and enzyme levels were calculated relative to tissue amounts (fmol mg^−1^).

### Colonic gene expression

RNA was extracted from snap-frozen colonic mouse tissue (Qiagen RNeasy mini kit cat # 74106, Venlo, The Netherlands) and RNA integrity and quantity was checked with Bioanalyzer 2100 (Agilent, Santa Clara, CA, USA). RNA sequencing and preparative techniques were performed by the Genomics Core (UZ Leuven, http://gc.uzleuven.be/). TruSeq stranded mRNA library preparation (Illumina, San Diego, CA, USA) was performed according to the manufacturer's guidelines. Sequencing was performed on the HiSeq2500 platform with a sequencing depth ranging from 9 to 21 M reads per sample (Illumina). The adaptors were trimmed from the reads with the use of ea_utils v1.1.2. Reads that were shorter than 25 bp after adaptor trimming were removed. The pre-processed reads were aligned to the reference genome of Mus musculus (Mm10) with TopHat v2.0.13. Counting of the reads was performed with HTSeq^46^ or CuffQuant^47^. Pathway analysis was performed with Ingenuity Pathway Analysis (IPA, Qiagen). Raw data have been deposited in BioProject with the accession code PRJNA374413 (https://www.ncbi.nlm.nih.gov/bioproject/374413).

### Treatment of DSS-induced colitis with peptide inhibitors

Acute colitis was induced in male and female mice via administration of 2% DSS in the drinking water for 7 days followed by 2–4 days on normal drinking water. Two peptide inhibitors (peptide inhibitor A (CPU1) and peptide inhibitor B (CPU2); Supplementary Table 2) were administered either daily via i.p. injections at a dose of 250 μg or continuously via implanted osmotic pumps at 30 mg kg^−1^ per day with a release rate of 0.25 μl per hour (pump type 1,002, DURECT Corporation, ALZET Osmotic Pumps, Cupertino, CA, USA). These doses were determined based on previously published structure activity relationships and *in vivo* dose-response data with these peptide inhibitors in another animal model of acute inflammation, namely lethal endotoxin shock in mice^16^. I.p. injections were given daily both in a therapeutic (starting at day 6) and prophylactic (starting at day 1) setting. Osmotic pumps were implanted subcutaneously on the back of the mice according to the manufacturer's guidelines. Briefly, osmotic pumps were pre-filled with 0.9% pyrogen-free NaCl, 100 μg μl^−1^ peptide inhibitor A or 100 μg μl^−1^ peptide inhibitor B and primed at 37 °C in 0.9% pyrogen-free NaCl for 1–2 h before implantation. Mice were sedated with the use of a mixture of Ketamine (100 mg ml^−1^) and Rompun (2%). The pre-filled and primed osmotic pumps were then implanted on the back of the mice, between and slightly posterior to the scapulae. The incision wound was closed with 7 mm wound clips (ALZET Osmotic Pumps, Cupertino, CA, USA) and a post-analgesic (Buprenorphine, 0.3 mg ml^−1^) was given subcutaneously. Mice were monitored and weighted every other day until completion of the study. Colonic inflammation and histopathology parameters were evaluated as described above.

### Quantitative RT-PCR

Snap-frozen mouse colonic fragments were mechanically homogenized with the use of Precellys 24 homogenizer (VWR, Leuven, Belgium). Total RNA was extracted according to the manufacturer's guidelines (RNeasy Mini kit, Qiagen, Hilden, Germany, #74104) and quantified with the use of a Nanodrop ND-1000 Spectrophotometer (Isogen Life Science, Temse, Belgium). cDNA was synthesized with the use of High-Capacity cDNA Reverse Transcription Kit (Applied Biosystems, Foster City, CA, USA). *Mmp3, Mmp8, Mmp9* and *Tace* mRNA levels were analysed in duplicate on 25 ng cDNA by quantitative RT-PCR with the use of TaqMan Universal PCR Master Mix from Applied Biosystems and primer and probe sets from Integrated DNA Technologies (Leuven, Belgium; Supplementary Table 3). Data were normalized to 18S ribosomal RNA levels^48^.

### Statistical analyses

Statistical analyses were performed using GraphPad Prism 5.03 (GraphPad, La Jolla, CA, USA). Data are represented as medians (interquartile range), unless otherwise stated, and *P* values were obtained using two-tailed Mann–Whitney *U* testing (*P<0.05, **P≤0.01, ***P≤0.001). Differences were considered statistically significant at *P*<0.05. For RNA sequencing data, statistical comparison of expression values was conducted with edgeR^49^, DESeq^50^ and CuffDiff2^51^. The resulting *P* values were corrected for multiple testing with Benjamini–Hochberg to control the FDR. To select significantly DE genes, a cut-off on FC to genes with an absolute log2-ratio larger than 2 was combined with genes with an FDR value <10%.

### Data availability

RNA sequencing data were generated at the Genomics Core, UZ Leuven. Data that support the findings of this study have been deposited in BioProject with the accession code PRJNA374413 (https://www.ncbi.nlm.nih.gov/bioproject/374413).

## Additional information

**How to cite this article:** de Bruyn, M. *et al*. Inhibition of gelatinase B/MMP-9 does not attenuate colitis in murine models of inflammatory bowel disease. *Nat. Commun.* **8,** 15384 doi: 10.1038/ncomms15384 (2017).

**Publisher's note:** Springer Nature remains neutral with regard to jurisdictional claims in published maps and institutional affiliations.

## Supplementary Material

Supplementary Information Supplementary figures and supplementary tables.

## Figures and Tables

**Table 1:** Overview of DE *Mmp* and *Timp* genes in the colon of MMP-9^−/−^ and WT mice in control and DSS conditions.

**Gene symbol**	**Gene name**	**DESeq**		**EdgeR**		**CuffDiff2**	
		**Log2 FC**	**Adj** ***P*** **value**	**Log2 FC**	**Adj** ***P*** **value**	**Log2 FC**	**Adj** ***P*** **value**
*MMP-9*^*−/−*^ *control (*n*=8) versus WT control (*n*=8)*							
Mmp9*	**Matrix metalloproteinase 9**	**3.9**	**7.89 e-34**	**3.8**	**3.20 e-40**	**3.9**	**0.019**
*Timp1*	Tissue inhibitor of metalloproteinase 1	0.1	1	0.1	1	0.1	1
							
*MMP-9*^*−/−*^ *DSS (*n*=8) versus WT DSS (*n*=8)*							
Mmp9*	Matrix metalloproteinase 9	−1.5	1	NA	NA	−1.3	0.999
*Timp1*	Tissue inhibitor of metalloproteinase 1	0.8	1	0.78	1	0.8	0.999
							
*WT DSS (*n*=8) versus WT control (*n*=8)*							
*Mmp2*	Matrix metalloproteinase 2	1.3	0.052	1.3	1.44 e-04	1.3	8.31 e-04
***Mmp3***	**Matrix metalloproteinase 3**	**6.8**	**2.76 e-04**	**6.8**	**3.04 e-24**	**6.8**	**8.31 e-04**
*Mmp7*	Matrix metalloproteinase 7	2.5	0.014	2.4	6.70 e-05	2.4	0.077
***Mmp8***^†^	**Matrix metalloproteinase 8**	**NA**	**1.90 e-04**	**10.8**	**3.58 e-23**	**NA**	**8.31 e-04**
***Mmp9***	**Matrix metalloproteinase 9**	**5.4**	**0.004**	**5.4**	**2.83 e-22**	**5.4**	**8.31 e-04**
***Mmp10***^†^	**Matrix metalloproteinase 10**	**5.4**	**0.004**	**5.4**	**2.83 e-22**	**6.2**	**8.31 e-04**
***Mmp12***	**Matrix metalloproteinase 12**	**6.2**	**4.05 e-05**	**6.2**	**1.89 e-24**	**2.0**	**8.31 e-04**
***Mmp13***	**Matrix metalloproteinase 13**	**5.3**	**0.002**	**5.3**	**5.20 e-18**	**5.3**	**8.31 e-04**
***Mmp19***	**Matrix metalloproteinase 19**	**2.3**	**0.019**	**2.3**	**4.07 e-06**	**2.3**	**0.005**
***Timp1***	**Tissue inhibitor of metalloproteinase 1**	**5.5**	**1.57 e-04**	**5.6**	**1.41 e-22**	**5.6**	**8.31 e-04**
							
*MMP-9*^*−/−*^ *DSS (*n*=8) versus MMP-9*^*−/−*^ *control (*n*=8)*							
*Mmp2*	Matrix metalloproteinase 2	4.2	0.368	2.1	1.45 e-04	2.1	0.018
*Mmp3*	Matrix metalloproteinase 3	7.9	0.118	NA	NA	7.9	0.003
*Mmp7*	Matrix metalloproteinase 7	3.5	0.031	3.5	5.44 e-06	3.5	0.325
***Mmp8***^†^	**Matrix metalloproteinase 8**	**11.9**	**9.37 e-06**	**10.6**	**1.17 e-23**	**11.9**	**0.009**
Mmp9*	Matrix metalloproteinase 9	1.05	1	NA	NA	0.2	0.871
***Mmp10***^†^	**Matrix metalloproteinase 10**	**8.0**	**0.005**	**8.0**	**1.95 e-20**	**8.0**	**7.61 e-04**
*Mmp12*	Matrix metalloproteinase 12	2.5	0.274	2.5	1.24 e-05	3.1	7.61 e-04
*Mmp13*	Matrix metalloproteinase 13	6.8	0.055	6.8	1.31 e-16	6.8	7.61 e-04
*Mmp19*	Matrix metalloproteinase 19	2.7	0.258	2.8	2.72 e-06	2.8	0.003
*Timp1*	Tissue inhibitor of metalloproteinase 1	6.2	0.079	6.3	1.51 e-15	6.3	7.61 e-04

adj, adjusted; DSS, dextran sodium sulphate; FC, fold change; NA, undetectable value.

^*^In MMP-9^−/−^ mice, reads were mapped only to exons 9–13 (non-functional read-through transcript containing the haemopexin domain), whereas no reads were mapped to exons 1–8 (the functional part of the *Mmp9* gene). This explains the paradoxical difference between MMP-9^−/−^ control and WT control mice.

Gene symbols and names in bold represent significance regarding differential expression, bold gene symbols with a dagger (†) indicate overlap between significantly DE genes.
