# Supplementary material for: Inhibition of gelatinase B/MMP-9 does not attenuate colitis in murine models of inflammatory bowel disease
Source: Nat Commun. 2017 May 31;8:15384. doi: 10.1038/ncomms15384 (PMC5460016; doi:10.1038/ncomms15384)
Supplement: Supplementary Information — Supplementary figures and supplementary tables. [file ncomms15384-s1.pdf]

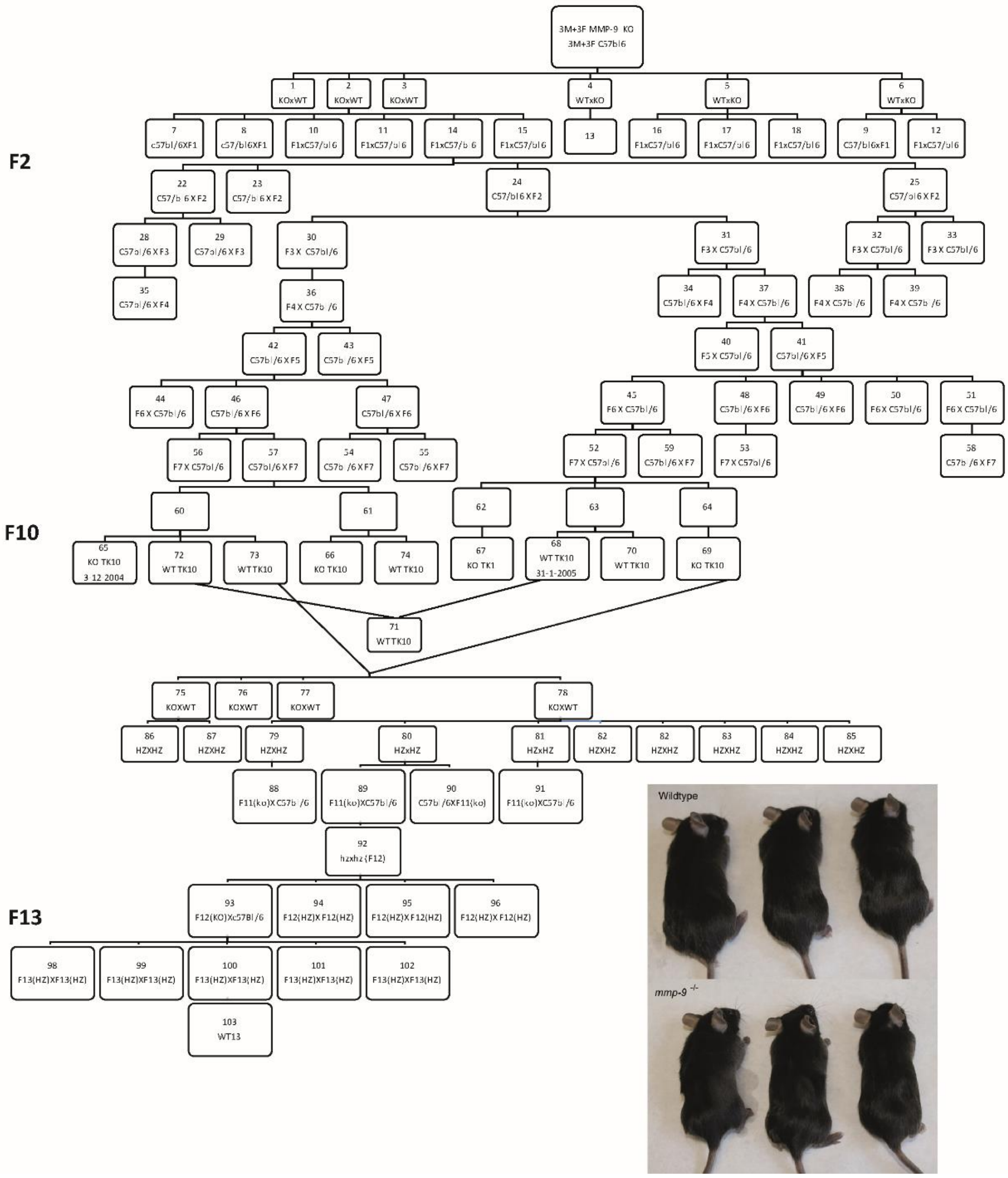

Supplementary figure 1: Backcrossing scheme of MMP-9$^{-/-}$ mice to C57BL/6J background for 13 generations and examples of three wild type and three MMP-9$^{-/-}$ mice.

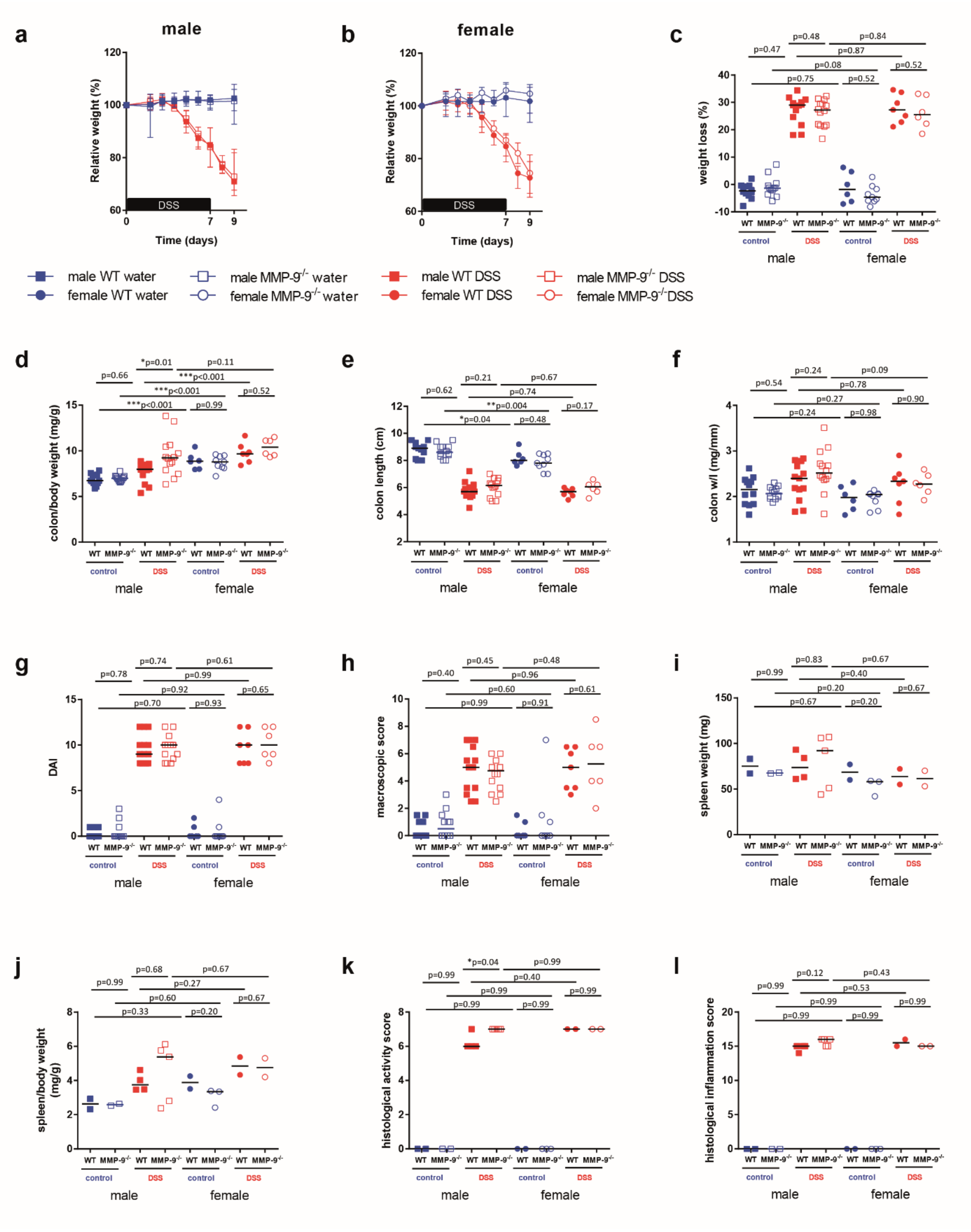

Supplementary figure 2: Differences between male (*squares*) and female (*circles*) mice after acute DSS administration. Data are shown from male control WT mice (n=12), male control MMP-9$^{-/-}$ mice (n=12), male DSS-treated WT mice (n=13), male DSS-treated MMP-9$^{-/-}$ mice (n=14), female control WT mice (n=6), female control MMP-9$^{-/-}$ mice (n=9), female DSS-treated WT mice (n=7) and female DSS-

treated MMP-9$^{-/-}$ mice (n=6) that were used in three independent experiments. Representation of relative body weight curves of male (**a**) and female (**b**) mice, absolute body weight loss at time of sacrifice (**c**), colon/body weight ratio (**d**), colon length (**e**), colon weight/length (w/l) ratio (**f**), disease activity index (DAI) (**g**), macroscopic damage score (**h**), spleen weight (**i**), spleen/body weight (**j**), histological activity score (**k**) and histological inflammation score (**l**). Median values with interquartile range, if applicable, are shown. Statistical analyses were performed with Mann-Whitney *U* tests (* $p<0.05$, ** $p\leq0.01$, *** $p\leq0.001$).

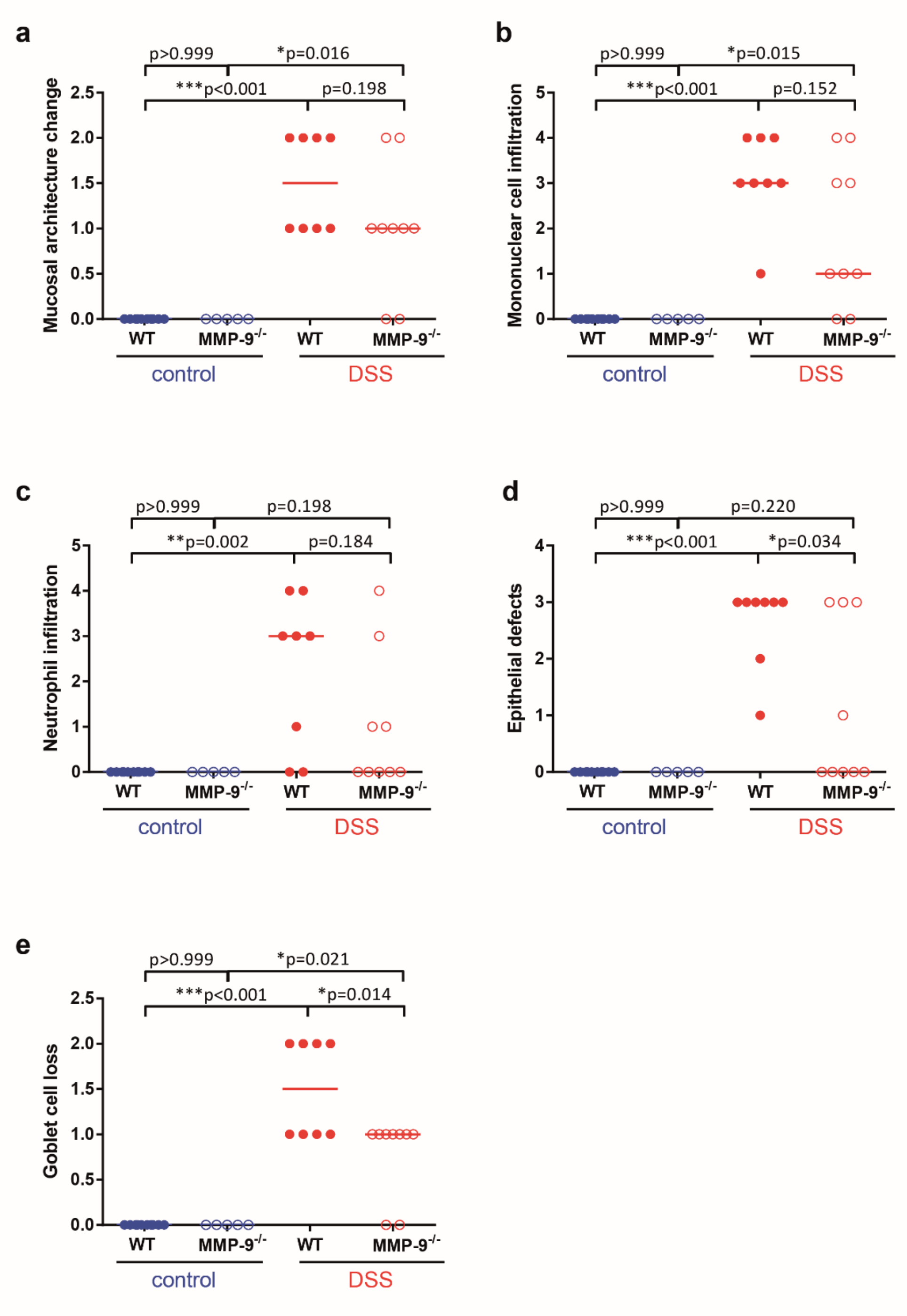

Supplementary figure 3: Histological inflammation and activity scores of WT and MMP-9$^{-/-}$ mice with chronic colitis. Read-outs from chronic DSS exposed WT (n=9) and MMP-9$^{-/-}$ mice (n=9), and control WT (n=10) and MMP-9$^{-/-}$ mice (n=5) included in three independent experiments are shown. Mucosal architecture change (**a**), mononuclear cell infiltration (**b**), neutrophil infiltration (**c**), epithelial defects (**d**) and goblet cell loss (**e**) are represented. Median values are indicated and statistical analyses were performed with Mann-Whitney *U* tests (* $p<0.05$, ** $p\leq0.01$, *** $p\leq0.001$).

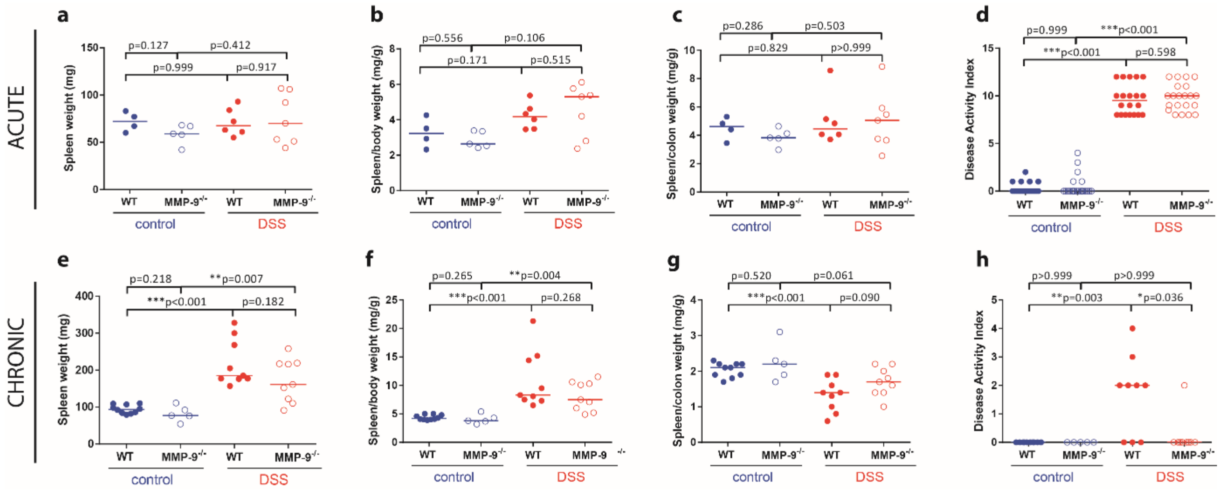

Supplementary figure 4: Systemic inflammation parameters of acute and chronic DSS models. Data are shown from acute DSS exposed WT (n=20) and MMP-9$^{-/-}$ mice (n=20) as well as control WT (n=18) and MMP-9$^{-/-}$ mice (n=21) for DAI measurements, whereas for spleen measurements a subset of samples was used (n=21). Pooled data from 3 replicated acute DSS experiments are shown. For the chronic model, read-outs from DSS exposed WT (n=9) and MMP-9$^{-/-}$ mice (n=9), and control WT (n=10) and MMP-9$^{-/-}$ mice (n=5) included in 3 independent experiments are shown. Spleen weight was evaluated as such and as a ratio over body weight and colon weight after acute (**a-c**) and chronic (**e-g**) DSS administration. The disease activity index (DAI) includes stool consistency, presence of blood and body weight loss and is represented for samples included in the acute (**d**) and chronic (**h**) DSS model. Median values are indicated and statistical analyses were performed with Mann-Whitney *U* tests (* p<0.05, ** p≤0.01, *** p≤0.001).

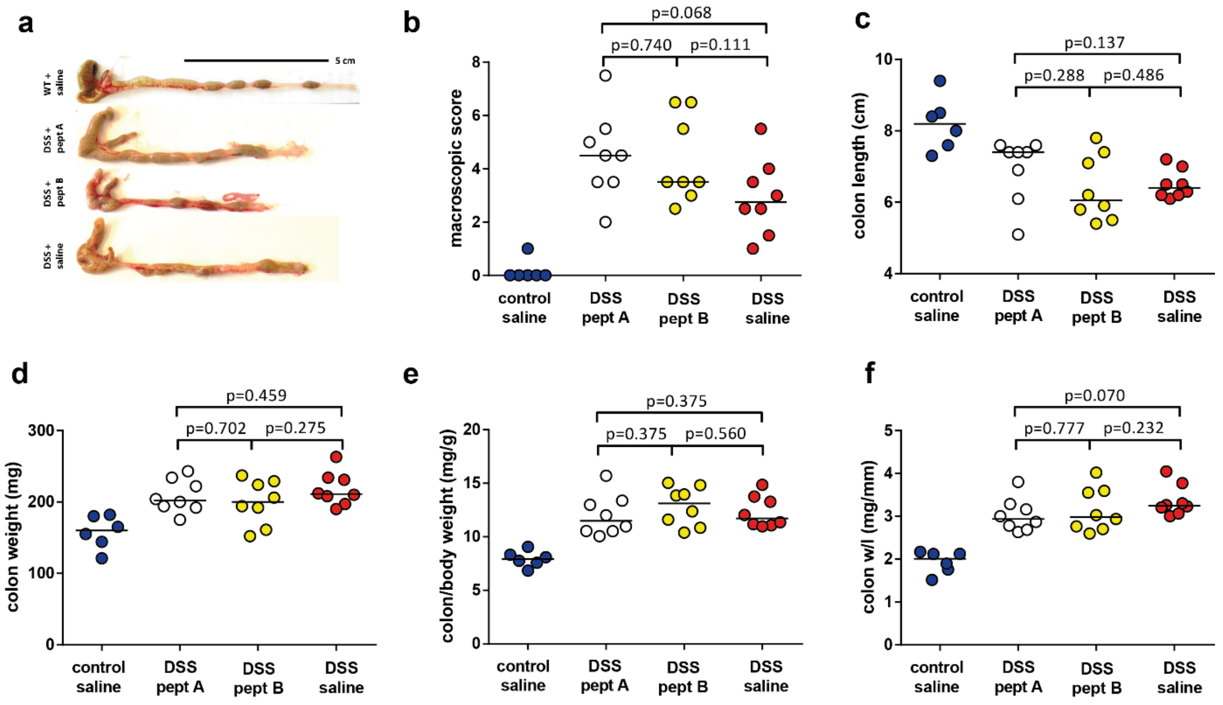

Supplementary figure 5: Macroscopic damage score and colonic measurements after therapeutic administration of peptide inhibitors A and B. Overview of isolated colon, cut at the ileocecal intersection isolated from WT + saline (n=6), DSS + peptide inhibitor A (n=8), DSS + peptide inhibitor B (n=8) and DSS + saline (n=8) mice (**a**). A scale bar of 5 cm is shown on the pictures. Representation of macroscopic damage score (**b**) colon length (**c**), colon weight (**d**), colon/body weight ratio (**e**) and colon weight/length (w/l) ratio (**f**) of mice treated therapeutically starting from day 6 with peptide inhibitors A, B or saline. Median values are represented and statistical analyses were performed with Mann-Whitney *U* tests.

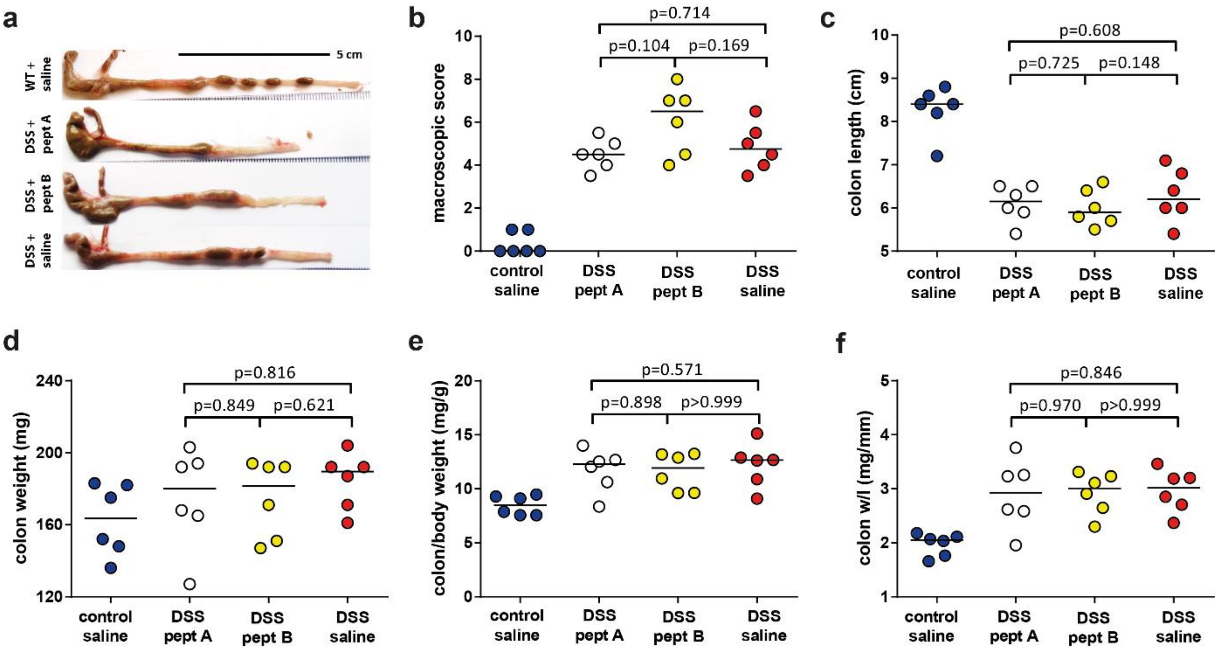

Supplementary figure 6: Macroscopic damage score and colonic measurements after prophylactic administration of peptide inhibitors A and B. Overview of isolated mouse colon, cut at the ileocecal intersection isolated from WT + saline (n=6), DSS + peptide inhibitor A (n=6), DSS + peptide inhibitor B (n=6) and DSS + saline mice (n=6) (**a**). A scale bar of 5 cm is shown on the pictures. Representation of macroscopic damage score (**b**), colon length (**a-c**), colon weight (**d**), colon/body weight ratio (**e**) or colon weight/length (w/l) ratio (**f**) of mice that received either peptide inhibitors A, B or saline after day 1 of start of DSS administration. Median values are represented and statistical analyses were performed with Mann-Whitney *U* tests.

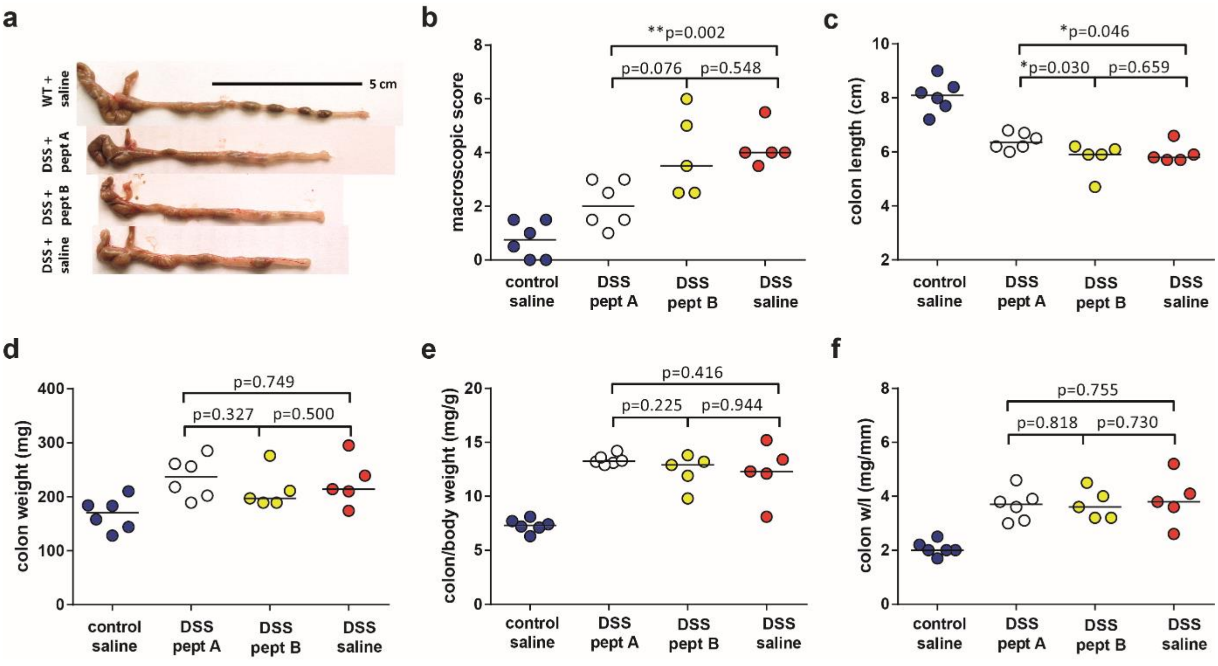

Supplementary figure 7: Macroscopic damage score and colonic measurements after continuous delivery of peptide inhibitors A and B via implanted osmotic pumps. Overview of isolated mouse colon, cut at the ileocecal intersection isolated from WT + saline (n=6), DSS + peptide inhibitor A (n=6), DSS + peptide inhibitor B (n=6) and DSS + saline mice (n=6) (**a**). A scale bar of 5 cm is shown on the pictures. Representation of macroscopic damage score (**b**), colon length (**a-c**), colon weight (**d**), colon/body weight ratio (**e**) or colon weight/length (w/l) ratio (**f**) of mice that were implanted with osmotic pumps containing either peptide inhibitors A, B or saline. Median values are represented and statistical analyses were performed with Mann-Whitney *U* tests (* $p<0.05$, ** $p\leq0.01$).

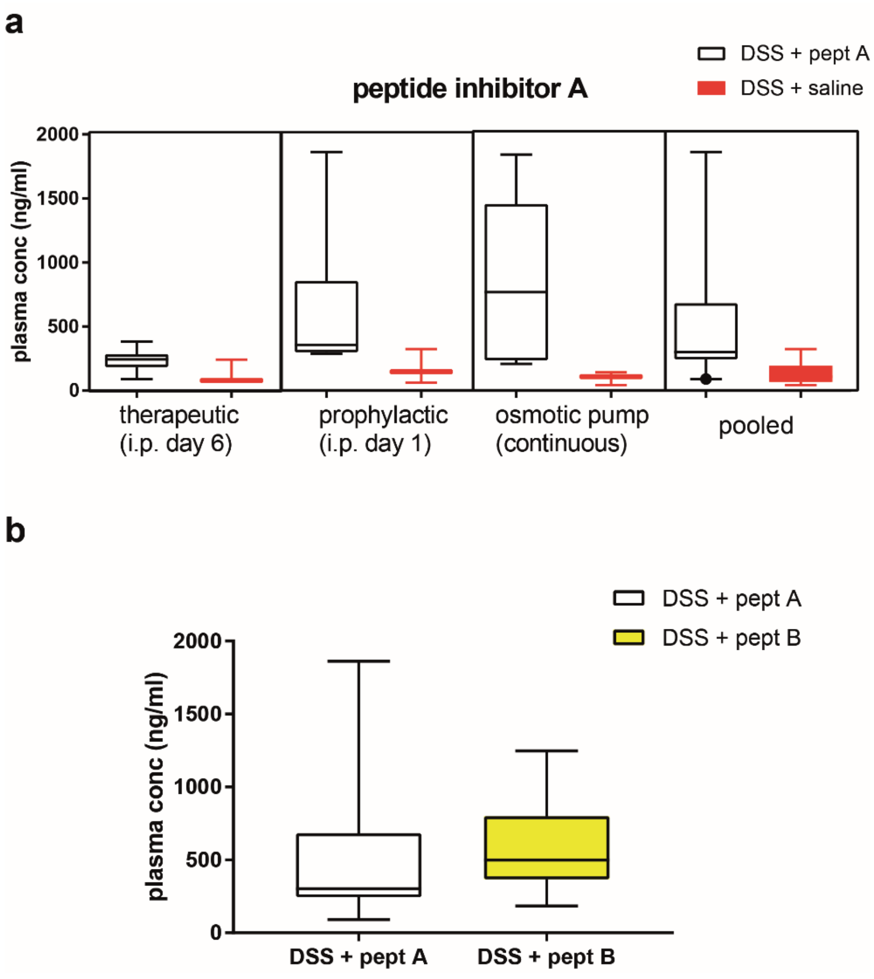

Supplementary figure 8: **Levels of peptide inhibitors A and B in plasma of DSS-treated mice at time of sacrifice.** (**a**) Plasma levels of peptide inhibitor A measured with ELISA in DSS-treated mice that received either peptide inhibitor A (n=19) or saline (n=9) injections for all three different administration schemes (therapeutic, prophylactic and continuous). In addition, pooled values are shown to indicate median detectable plasma levels regardless of the administration scheme. (**b**) Similar ELISA analyses were performed for detection of peptide inhibitor B in plasma of DSS-treated mice (n=19). This indicates that both peptide inhibitors A and B yield detectable circulating plasma levels at time of sacrifice confirming that sufficiently high levels for inhibition in plasma were attained. Colour code is similar as in Figures 9 and 10. Box plots are shown with 5-95 percentiles.

SUPPLEMENTARY TABLES

Supplementary table 1: Overview of significantly differentially expressed genes in the colon between control and DSS-treated WT and MMP-9$^{-/-}$ mice after DESeq, EdgeR and CuffDiff2 comparative analyses. Abbreviations: FC, fold change; adj, adjusted; DSS, dextran sodium sulphate; NA, undetectable value.

Gene symbol	Gene name	DESeq		EdgeR		CuffDiff2	
		Log2 FC	Adj p-value	Log2 FC	Adj p-value	Log2 FC	Adj p-value
MMP-9$^{-/-}$ control (n=8) *versus* WT control (n=8)							
Mmp9†	matrix metalloproteinase 9	3.9	7.89 e-34	3.8	3.20 e-40	3.9	0.019
Rims4	regulating synaptic membrane exocytosis 4	-6.0	3.89 e-12	-6.0	5.65 e-37	-6.0	0.019
Slpi	secretory leukocyte peptidase inhibitor	2.4	1	2.4	3.68 e-04	2.5	0.019
MMP-9$^{-/-}$ DSS (n=8) *versus* WT DSS (n=8)							
Clps	colipase, pancreatic	NA	NA	5.6	0.020	5.7	0.030
Ddx60	DEAD (Asp-Glu-Ala-Asp) box polypeptide 60	NA	NA	2.4	0.033	2.4	0.030
Fgb	fibrinogen beta chain	NA	NA	7.6	0.020	7.7	0.030
Fgg	fibrinogen gamma chain	NA	NA	7.5	0.020	7.6	0.030
Ifi44	interferon-induced protein 44	NA	NA	2.5	0.020	2.1	0.030
Ifit2	interferon-induced protein with tetratricopeptide repeats 2	NA	NA	2.6	0.011	2.6	0.030
Isg15	ISG15 ubiquitin-like modifier	NA	NA	2.4	0.020	2.4	0.030
Itih3	inter-alpha-trypsin inhibitor heavy chain 3	NA	NA	4.1	0.029	4.2	0.030
Itih4	inter-alpha-trypsin inhibitor heavy chain family, member 4	NA	NA	7.0	0.003	6.3	0.030
Oas3	2'-5'-oligoadenylate synthetase 3, 100kDa	NA	NA	2.7	0.020	2.7	0.030
Usp18	ubiquitin specific peptidase 18	NA	NA	2.5	0.020	2.5	0.030

† In MMP-9$^{-/-}$ mice, reads were mapped only to exons 9-13 (non-functional read-through transcript containing the haemopexin domain), whereas no reads were mapped to exons 1-8 (the functional part of the *Mmp9* gene). This explains the paradoxical difference between MMP-9$^{-/-}$ control and WT control mice.

Supplementary table 2: IC50 values of peptide inhibitors A and B towards MMP-3, -8, -9 and TACE (μM).

Name	Peptide sequences†	MMP-3	MMP-8	MMP-9	TACE
Peptide inhibitor A (CPU 1)	P(Pyr)CBRGE-GGGG-IVRRADRAAVP	No inhibition	17.6	12.5	7.0
Peptide inhibitor B (CPU 2)	IVRRADRAAVP-GGGG-P(Pyr)CBRGE	7.0	0.036	0.16	15

† Peptide sequences are in one-letter codes for amino acids. Pyr stands for pyridylalanine.

Supplementary table 3: Primer sequences used for qRT-PCR analyses.

Gene	Forward primer	Reverse primer	Probe
Mmp3	CAGGAAGATAGCTGAGGACTTTC	GGTCAAATTCCAACTGCGAAG	AGGTGTTGACTCAAGGGTGGATGC
Mmp8	CCAGCACCTATTCACTACCTC	AGCATCAAATCTCAGGTGGG	ACCTTCAGACAACCCCATCCAACC
Mmp9	GATCCCCAGAGCGTCATTC	CCACCTTGTTCACCTCATTTTG	CGACATCTTCCAGTACCAAGACAAAGCC
Tace	GGGTTTTGCGACATGAATGG	GAAAACCAGAACAGACCCAAC	TCCCAAAATCGCTCAATTACGTCCTGT
